# Estimating Leaf CO_2_ Assimilation in C_3_ Plants Using a Handheld Porometer With Chlorophyll Fluorometer in Field Conditions

**DOI:** 10.1111/pce.70006

**Published:** 2025-06-18

**Authors:** Kensuke Kimura, Erina Fushimi, Etsushi Kumagai, Koichi Nomura, Toshinori Matsunami, Shohei Konno, Atsushi Maruyama

**Affiliations:** ^1^ Institute for Agro‐Environmental Sciences National Agriculture and Food Research Organization (NARO) Tsukuba Japan; ^2^ IoP Collaborative Creation Center Kochi University Nankoku Japan; ^3^ Tohoku Agricultural Research Center National Agriculture and Food Research Organization (NARO) Morioka Japan; ^4^ Institute of Fruit Tree and Tea Science National Agriculture and Food Research Organization (NARO) Tsukuba Japan

**Keywords:** chlorophyll fluorescence, gas exchange, herbaceous plants, high‐throughput phenotyping, photosynthesis, pulse amplitude modulated (PAM) fluorometry, stomatal conductance, sunlight, woody plants

## Abstract

Gas exchange measurement is the gold standard method for determining leaf CO_2_ assimilation rate (*A*_n_). However, conventional systems for measuring *A*_n_ often require time and/or effort to collect numerous samples in the field owing to their high weight and large size. Here, we present an efficient and convenient method for estimating *A*_n_ using a handheld porometer with a chlorophyll fluorometer, facilitating on‐the‐go assessment of *A*_n_ in the field. This porometer‐fluorometer method integrates the measured stomatal conductance and quantum yield of photochemistry in PSII into a biochemical photosynthesis model, incorporating model uncertainties into a single calibrated parameter. Using this method, we successfully estimated *A*_n_ variations in 12 species under field conditions, with a root mean square error of 2.0 μmol m^−2^ s^−1^, despite using the common parameter set. In contrast, without calibration (i.e., with the often‐assumed parameter value), this method greatly overestimated *A*_n_. These results highlight the importance of appropriate calibration depending on prevailing conditions, particularly the light source. In summary, this method demonstrates the potential for accessible, high‐throughput, and accurate estimation of *A*_n_ in diverse plants, thereby addressing a key bottleneck in field‐based phenotyping of photosynthesis. However, further studies are required to reduce the uncertainties imposed on the calibrated parameter.

## Summary statement

This study presents an efficient and convenient method for estimating leaf CO_2_ assimilation rate (*A*_n_) using a handheld porometer with a chlorophyll fluorometer, facilitating on‐the‐go assessment of *A*_n_ in the field.

## 1 Introduction

Photosynthesis is essential for plant growth and development and provides critical insights into plant health and productivity. In agriculture, understanding photosynthetic performance is valuable for breeding crops and trees with enhanced photosynthetic efficiency and yield potential (Zhu et al. 2010; Simkin et al. 2019). Gas exchange measurement is the gold standard for measuring leaf photosynthesis (i.e., leaf CO_2_ assimilation rate, *A*_n_). Conventional systems using an assimilation chamber and infrared gas analyzers can provide detailed photosynthetic information, such as the maximum rates of RuBisCO carboxylation and electron transport, owing to the precise environmental control of these systems (Saathoff and Welles 2021). However, their large size and high weight (~10 kg) demand significant effort to hold them throughout the measurement, making them impractical for on‐the‐go field measurements, particularly when moving across multiple leaves in a large field. Although tripods can reduce the effort required to fix the equipment, setting up a tripod for each measurement is time‐consuming. Moreover, gas exchange systems require stabilization time in minutes, raising the total time for each measurement to at least several minutes. Such effort and time considerations may make researchers, particularly who need to screen numerous samples in a large field (such as breeding programs in agriculture), refrain from gas exchange measurements and miss opportunities to phenotype photosynthesis. Therefore, there is a need for more accessible and high‐throughput techniques than conventional systems.

Recently, a handheld porometer equipped with a pulse‐amplitude modulated (PAM) chlorophyll fluorometer was developed (e.g., LI‐600 from LI‐COR bioscience), offering a practical tool for measuring critical photosynthetic parameters, including stomatal conductance (*g*_s_) and quantum yield of photochemistry in PSII (Φ_PSII_). This compact and lightweight (~1 kg) device can simultaneously measure *g*_s_ and Φ_PSII_ within seconds, making it well‐suited for high‐throughput field phenotyping and significantly easier to use for on‐the‐go assessment than traditional gas exchange systems. Although these instruments do not directly measure leaf CO₂ assimilation (*A*_n_), He and Edwards (1996) proposed a mechanistic model that estimates *A*_n_ using *g*_s_ and Φ_PSII_ as inputs. This model was developed based on the biochemical processes of photosynthesis and is applicable to C_3_ plants. Although the original model requires an empirical model to estimate *g*_s_ (Leuning 1995), modern porometers can directly measure *g*_s_; thus, a more accurate estimation of *A*_n_ is expected (Kitao et al. 2021). Therefore, integrating handheld instruments with mechanistic models offers an accessible, high‐throughput, and accurate approach to estimating *A*_n_ in diverse C_3_ species.

Despite the potential of this porometer‐fluorometer approach, its practical application is limited by the uncertainties in estimating the electron transport rate (*J*) within the model. *J* is often estimated using Φ_PSII_ measured by a chlorophyll fluorometer, as follows (Genty et al. 1989):

(1) $$J\;=\;\Phi_{\text{PSII}}\;\text{PPFD}\;\alpha \;\beta ,$$

where PPFD is the photosynthetic photon flux density, *α* is the leaf absorptance of PPFD, and *β* is the fraction of the absorbed light allocated to PSII. Typically, *α* and *β* are set at 0.84 (or 0.85) and 0.5, respectively, although these values likely differ among species (Laisk and Loreto 1996) and growth light conditions (Chow et al. 1990; Murakami et al. 2018). Some studies have suggested additional parameters in Equation (1) to correct for *J* overestimations due to light absorption by non‐photosynthetic pigments (Loreto et al. 2009; McClain and Sharkey 2020), alternative electron sinks (Yin et al. 2009), and mesophyll layer heterogeneity (Evans 2009). These errors are remarkable under high blue‐to‐red light ratios, such as natural sunlight (McClain and Sharkey 2020), leading many studies to use artificial light with a minimal blue light to minimize *J* overestimation. Consequently, few studies have examined *J* estimation under sunlight, the most common light source for plant growth, leaving the necessary corrections across species and growth conditions unclear.

In this study, we tested whether combining a handheld porometer with a chlorophyll fluorometer and a biochemical photosynthesis model could accurately estimate *A*_n_ variations under natural sunlight across 12 C₃ species grown in varied conditions. We assessed how empirical calibration affected estimation error correction and discussed the potential for field applications of this method. This approach offers a practical solution to the bottleneck in phenotyping photosynthesis under field conditions, enabling more accessible and accurate *A*_n_ assessments in diverse environments.

## 2 Materials and Methods

### 2.1 Model for Estimating Leaf CO_2_ Assimilation

The relationship between *A*_n_ and *J* was formulated based on the electron requirements for carboxylation and oxygenation using the biochemical photosynthesis model of Farquhar et al. (1980) as follows:

(2) $$A_{n}=\;\frac{J\;(C_{i}-\Gamma^{*})}{4(C_{i}\;+\;{2\Gamma }^{*})}-R_{d},$$

where *C*_i_ is the intercellular CO_2_ concentration, Γ* is the CO_2_ compensation point in the absence of day respiration, and *R*_d_ is day respiration rate. Although *A*_n_ is limited by either RuBisCo, ribulose 1,5‐bisphosphate (RuBP) regeneration, or triose phosphate utilization (TPU) (Farquhar et al. 1980; Sharkey 1985), Equation (2) can be applied to all three limitation phases when “actual” *J* is directly quantified (Harley et al. 1992). *J* was quantified by modifying Equation (1) after Loreto et al. (2009), Yin et al. (2009), and van der Putten et al. (2018) are as follows:

(3) $$J=\Phi_{\text{PSII}}\;\text{PPFD}\;\alpha \;\beta \;\gamma \;\left(1-\frac{f_{\text{pseudo}}}{1-f_{\text{cyc}}}\right)\xi =\Phi_{\text{PSII}}\;\text{PPFD}\;s,$$

where *γ* is the correction factor accounting for the light absorption by non‐photosynthetic pigments (Loreto et al. 2009; McClain and Sharkey 2020); *f*_pseudo_ and *f*_cyc_ are the fractions of total electron flux used as pseudo‐cyclic and cyclic electron transport in PSI, respectively (Yin et al. 2009), representing alternative electron flows; and *ξ* is the ratio of true Φ_PSII_ and measured Φ_PSII_ accounting for measurement errors of Φ_PSII_ (van der Putten et al. 2018) owing to mesophyll layer heterogeneity (Evans 2009). These parameters, in addition to *α* and *β*, are difficult to determine under field conditions; thus, we lumped these hard‐to‐measure parameters as the calibrated parameter *s* (Yin et al. 2009; van der Putten et al. 2018).

*C*_i_ was calculated by the CO_2_ transfer of the leaf as follows:

(4) $${C_{i}=C}_{a}-\frac{A_{n}}{g_{\text{tc}}},$$

where *C*_a_ is the ambient air CO_2_ concentration and *g*_tc_ is the leaf total conductance for CO_2_ transfer on both leaf sides. Porometers generally measure the conductance for one side of a leaf, and thus the measurement of the adaxial and abaxial sides of the leaf is required to calculate *g*_tc_. According to the electrical circuit theory described by *Ohm's law*, the conductances of the leaf boundary layer and stomata are connected in series, whereas those for the adaxial and abaxial sides of the leaf are parallel. This results in the calculation of *g*_tc_ as follows:

(5) $$g_{\text{tc}}={\;(1.37g_{\text{bw},\text{ad}}^{-1}\;+\;{1.6\;g}_{\text{sw},\text{ad}}^{-1})}^{-1}\;+{\left(1.37g_{\text{bw},\text{ab}}^{-1}\;+\;{1.6\;g}_{\text{sw},\text{ab}}^{-1}\right)}^{-1},$$

where *g*_bw_ and *g*_sw_ are the conductance of the leaf boundary layer and stomata for water vapor transfer, respectively, and the subscripts ad and ab represent the adaxial and abaxial sides of the leaf, respectively. The coefficients 1.37 and 1.6 are the conversion factors from water vapor to CO_2_ diffusivity.

Γ* was determined as follows:

(6) $$\Gamma^{*}=\;\frac{0.5\;O}{S_{c/o}},$$

where *O* is the oxygen concentration (210 mmol mol^−1^ in this study), *S*_c/o_ is the RuBisCO specificity factor, and the temperature response of *S*_c/o_ was determined using the Arrhenius equation, as follows:

(7) $$S_{c/o}=\;S_{c/o,25}\;\text{exp}\left[\frac{E_{a,S_{c/o}}\left(T_{l,K}-298.15\right)}{298.15\;R\;T_{l,K}}\right],$$

where *S*_c/o,25_ is S_c/o_ at 25°C, $E_{a,S_{c/o}}$ is the activation energy of *S*_c/o_ to temperature, *R* is the universal gas constant (8.314 J mol^−1^ K^−1^), *T*_l_ is the leaf temperature, and the subscript K represents kelvin unit.

*R*_d_ was determined using the Arrhenius equation, as follows:

(8) $$R_{d}=R_{d,25}\;\text{exp}\left[\frac{E_{a,R_{d}}\left(T_{l,K}-298.15\right)}{298.15\;R\;T_{l,K}}\right],$$

and *R*_d,25_ is *R*_d_ at 25°C, $E_{a,R_{d}}$ is the activation energy of *R*_d_ to temperature.

*A*_n_ can be estimated by Equations (2) to (8) using measurable variables of Φ_PSII_, PPFD, *g*_bw_, *g*_sw_, *C*_a_, and *T*_l_ along with parameters of *S*_c/o,25_, $E_{a,S_{c/o}}$, *R*_d,25_, $E_{a,R_{d}}$, and *s*. The analytical solution for *A*_n_ is described in Supporting Information S1: Note S1, and a template file for *A*_n_ estimation is provided in Supporting Information S2. In this study, *S*_c/o,25_ and $E_{a,S_{c/o}}$ were set to 2.57 mmol mol^−1^ and –28550 J mol^−1^, respectively, which were re‐calculated based on the In Vitro average values of C_3_ plants (Galmés et al. 2016). *R*_d,25_ and $E_{a,R_{d}}$ were set to 1.27 μmol m^−2^ s^−1^ and 59170 J mol^−1^, based on the average values of wheat under photorespiratory conditions (Fang et al. 2022). Thus, *s* was the sole calibrated parameter in this method, and uncertainties in other parameters, as well as those not explained by this model (e.g., mesophyll conductance, *g*_m_), were imposed on the calibrated *s*. Although *s* is generally calibrated under low O_2_ conditions to remove the influence of photorespiration (i.e., *C*_i_ and Γ*), this cannot calibrate the uncertainties of *C*_i_ and Γ* and requires further calibration to accurately estimate *A*_n_. Moreover, changing the O_2_ level is time‐consuming in the field. Therefore, we calibrated *s* under ambient O_2_ conditions to avoid multiple calibrations and to maintain the high‐throughput capability of the proposed method for field applications. The detailed processes of the calibration are described in Section 2.5.

### 2.2 Plant Materials

We selected six herbaceous and six woody plants that were grown under sunlight at different experimental sites in Japan. Among the six herbaceous species, wheat (*Triticum aestivum*) and soybean (*Glycine max*) were grown outdoors, whereas tomato (*Solanum lycopersicum*), eggplant (*Solanum melongena*), bell pepper (*Capsicum annuum*), and spinach (*Spinacia oleracea*) were grown in greenhouses. All six woody species [apple (*Malus domestica*), peach (*Prunus persica*), pear (*Pyrus pyrifolia*), loquat (*Rhaphiolepis bibas*), lemon (*Citrus limon*), and orange (*Citrus unshiu*)] were grown outdoors. We used three cultivars of soybean and one cultivar for each of the other species; thus, 14 cultivars from the 12 species were used in this study. The detailed growth conditions are described in Supporting Information S1: Note S2.

### 2.3 Measurements of Leaf Gas Exchange and Chlorophyll Fluorescence

**Figure 1:**
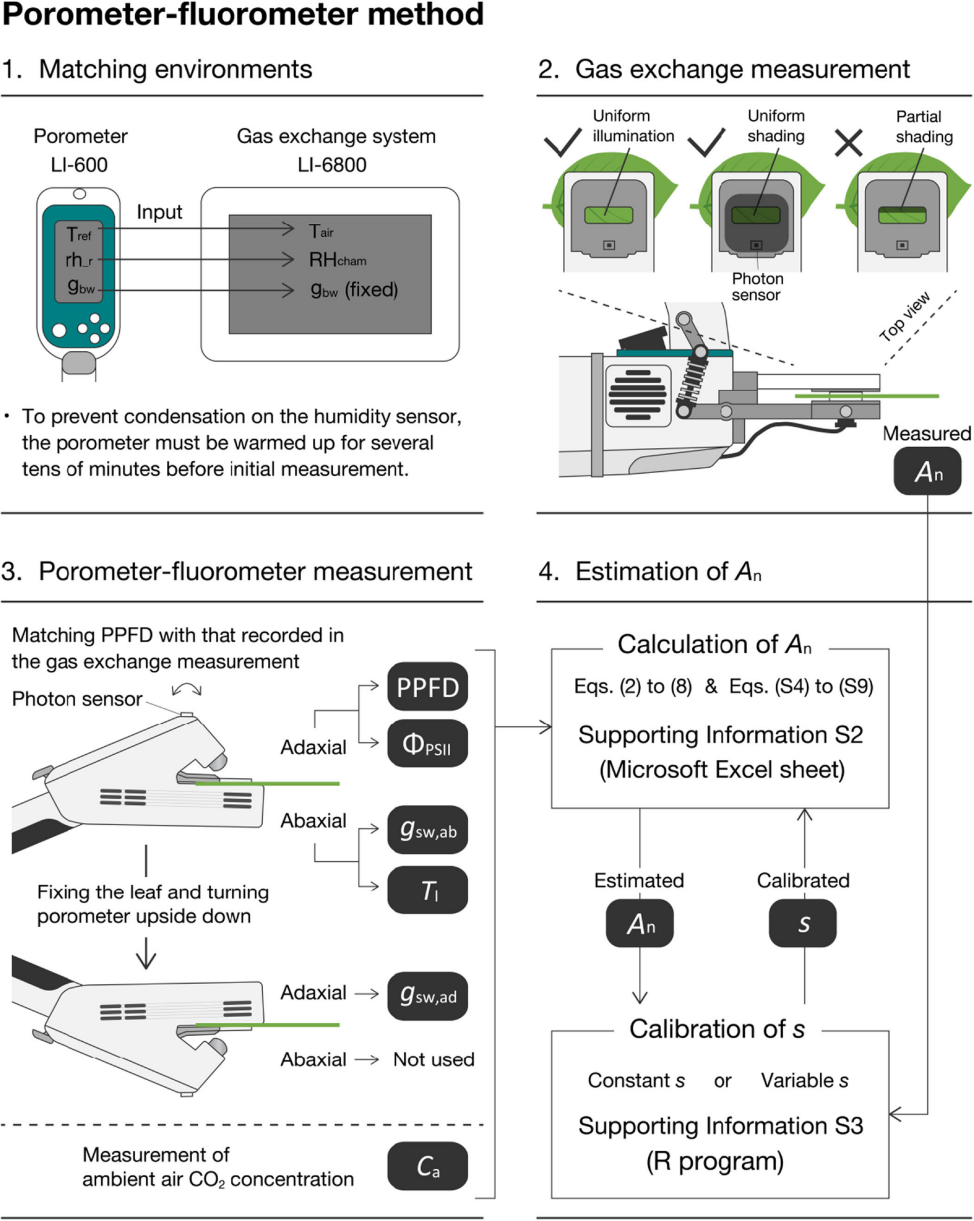
Procedure for estimating leaf CO_2_ assimilation rate (*A*_n_) using the porometer‐fluorometer method. See Section 2 for detailed explanations.

To test the porometer‐fluorometer method, gas exchange and chlorophyll fluorescence of leaves were measured in the field under sunlight using a gas exchange measurement system (LI‐6800; Li‐Cor, USA) and a porometer equipped with a chlorophyll fluorometer (LI‐600; Li‐Cor, USA). During the day, *A*_n_ was measured on a fully expanded leaf using the LI‐6800 with a clear top chamber to capture natural sunlight, and with a 1 × 3 cm^2^ chamber aperture plate to clamp thin leaves and avoid heterogeneous distribution of *A*_n_ on the measurement area as much as possible. After clamping the leaf with the adaxial side facing up, the measurement area faced toward the sun by changing the angle of the chamber head to avoid partial shading of the measurement area. During the measurement, the air temperature (*T*_a_) and relative humidity (*h*) in the LI‐6800 system were maintained at values monitored on the LI‐600 porometer to match the environmental conditions between the instruments. *g*_bw_ in the LI‐6800 system was maintained at approximately 2.9 H_2_O mol m^−2^ s^−1^, which is equivalent to the value in the LI‐600 porometer, by adjusting the fan speed in the chamber. *C*_a_ in the LI‐6800 system was also maintained at ambient values measured using a nondispersive infrared sensor (TR‐76Ui, T&D, Japan). Other commercialized porometers (e.g., MINI‐PAM‐II/POROMETER; WALZ) can simultaneously measure *C*_a_ and perform an estimation independently. The data were logged once *A*_n_ and *g*_sw_ in the LI‐6800 system stabilized. Immediately after removing the leaf from the chamber, *g*_sw_, *g*_bw_, PPFD, Φ_PSII_, and *T*_l_ were measured using the LI‐600 porometer equipped with the fluorometer. A multiphase flash method (Loriaux et al. 2013) was used to measure Φ_PSII_. The measurement was first performed on the adaxial side of the leaf, with PPFD values matching those recorded in the LI‐6800 system, and repeated three times along the measurement area of *A*_n_. The same procedure was then conducted on the abaxial side while maintaining the angle and orientation of the leaf (i.e., with the LI‐600 porometer upside down). These measurements were repeated for different leaves of each species and cultivar over 21 days (January to November 2024). The procedure pertaining to the porometer‐fluorometer method is summarized in Figure 1.

### 2.4 Data Analysis and Quality Control

*J* in Equation (3) was calculated using the mean values of Φ_PSII_ and PPFD measured on the adaxial side illuminated by the sun (i.e., we did not use Φ_PSII_ and PPFD on the abaxial side: Figure 1). The *g*_tc_ in Equation (5) was calculated using the mean values of *g*_bw_ and *g*_sw_ measured on each leaf side (Figure 1). Γ* and *R*_d_ in Equations (6) and (7), respectively, were calculated using the mean values of *T*_l_ from the abaxial side (Figure 1).

Although air temperature and relative humidity did not vary abruptly during each measurement period (lasting several minutes to tens of minutes), light conditions occasionally fluctuated between the measurements of the LI‐6800 system and the LI‐600 porometer because of the sudden cloud cover or sun beam. We excluded data recorded under such light conditions and finally used 310 samples for the following analysis. These samples included a wide range of environmental and physiological conditions (Supporting Information S1: Figure S1) and were considered sufficient for model construction and validation.

### 2.5 Calibration and Validation for Estimating Leaf CO_2_ Assimilation

To assess the accuracy and applicability of the porometer‐fluorometer method for estimating *A*_n_, cross‐validation was performed while empirically calibrating the lumped parameter *s* in Equation (3) using the following two approaches:

1. Constant *s*: Constant *s* is valid under low‐light conditions, particularly with artificial light (e.g., Loreto et al. 2009; van der Putten et al. 2018). Therefore, we firstly tested *A*_n_ estimation using a constant *s*. First, all the data were divided into 14 datasets for each cultivar. Then, *s* was calibrated on all the samples except for one data set, and *A*_n_ was calculated for the left‐out data set using the calibrated *s*. This process was repeated 14 times until all datasets were tested. The calibration of *s* was performed by minimizing the root mean square error (RMSE) between the estimated and observed values of *A*_n_ using differential evolution with the DEoptim package in R.
2. Variable *s*: McClain and Sharkey (2020) indicated that *s* decreases with an increase in *J*. Therefore, we tested *A*_n_ estimation using a variable *s* as a function of Φ_PSII_ × PPFD, which serves as a proxy for *J*. The same cross‐validation described above was applied, whereas *s* was calibrated using the following empirical equation:

(9) $$s=\frac{a_{s}}{1+\text{exp}\left[b_{s}\left(\Phi_{\text{PSII}}\text{PPFD}-c_{s}\right)\right]}+d_{s},$$

where a_s_, b_s_, c_s_, and d_s_ are the calibrated parameters. This equation shows a decreasing sigmoid curve from a_s_ + d_s_ (upper limit) to d_s_ (lower limit). The parameter b_s_ is related to the decreasing rate of s, and c_s_ is the value of Φ_PSII_ PPFD when s is a_s_/2 + d_s_. These parameters were calibrated by minimizing the RMSE between the estimated and observed values of A_n_ using the differential evolution described above. The R program for calibration and cross‐validation is provided in Supporting Information S3.

These two validation methods were designed to assess the applicability of the common parameters across various species and cultivars. The use of common parameters minimizes the calibration frequency and enhances the efficiency of *A*_n_ estimation.

## 3 Results

**Figure 2:**
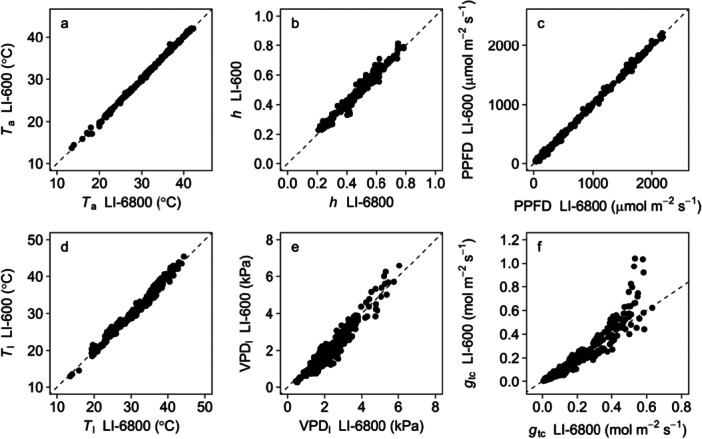
Comparison of (a) air temperature (*T*_a_), (b) relative humidity (*h*), (c) photosynthetic photon flux density (PPFD), (d) leaf temperature (*T*_l_), (e) leaf vapor pressure deficit (VPD_l_), and (f) leaf total conductance for CO_2_ transfer (*g*_tc_) between the gas exchange measurement system (LI‐6800) and the porometer equipped with the chlorophyll fluorometer (LI‐600).

*T*_a_, *h*, and PPFD in the LI‐6800 system were closely aligned with those in the LI‐600 porometer, showing a minimal deviation in environmental conditions between the two instruments (Figure 2a–c). *T*_l_ and leaf vapor pressure deficit (VPD_l_), which strongly affect the physical and physiological processes of leaf photosynthesis, were also similar between the two instruments (Figure 2d,e). The *g*_tc_ determined from the adaxial and abaxial values of *g*_sw_ and *g*_bw_ measured using the LI‐600 porometer were similar to the both‐sided *g*_tc_ determined from the LI‐6800 system, although an overestimation was observed at high *g*_tc_ values above 0.5 mol m^−2^ s^−1^ (Figure 2f). Although the cause of this overestimation requires further investigation, this error has a limited impact on the *A*_n_ estimation because *A*_n_ is primarily constrained by other factors when *g*_tc_ is significantly high (Supporting Information S1: Figure S2).

**Figure 3:**
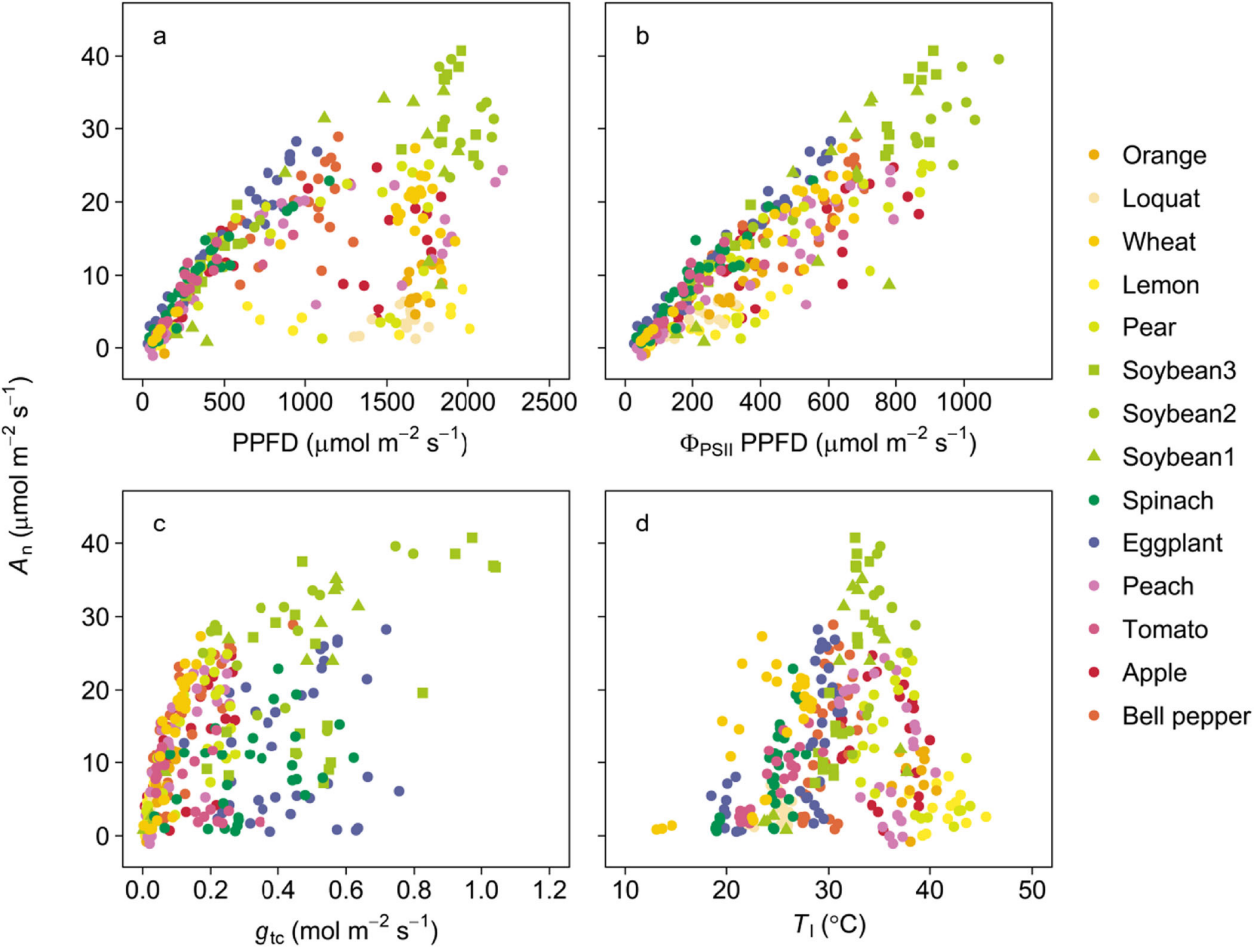
Relationship between leaf CO_2_ assimilation rate (*A*_n_) and (a) photosynthetic photon flux density (PPFD), (b) quantum yield of photochemistry in PSII (Φ_PSII_) × PPFD, which is the proxy of electron transport rate, (c) leaf total conductance for CO_2_ transfer (*g*_tc_), and (d) leaf temperature (*T*_l_) in 14 cultivars among 12 species. *A*_n_ was measured by the gas exchange measurement system (LI‐6800), and Φ_PSII_, PPFD, *g*_tc_, and *T*_l_ were inferred from the porometer equipped with the chlorophyll fluorometer (LI‐600). [Color figure can be viewed at wileyonlinelibrary.com]

The relationships between *A*_n_ and PPFD, Φ_PSII_PPFD, *g*_tc_, and *T*_l_ were nonlinear and species‐specific (Figure 3). *A*_n_ increased with PPFD at low PPFD, but became saturated at high PPFD, with saturation points varying by species (Figure 3a). Although *A*_n_ increased with Φ_PSII_PPFD, the slope of this relationship varied across species (Figure 3b). *A*_n_ increased with *g*_tc_ at low *g*_tc_ (< 0.2 mol m^−2^ s^−1^), but saturated at higher *g*_tc_, with saturation points varying by species (Figure 3c). The relationship between *A*_n_ and *T*_l_ showed peaks at a species‐specific optimal temperature (Figure 3d). These findings indicate that the individual variables measured by the LI‐600 porometer and fluorometer alone cannot provide accurate *A*_n_ estimates across different environments and species (Supporting Information S1: Figure S3).

**Figure 4:**
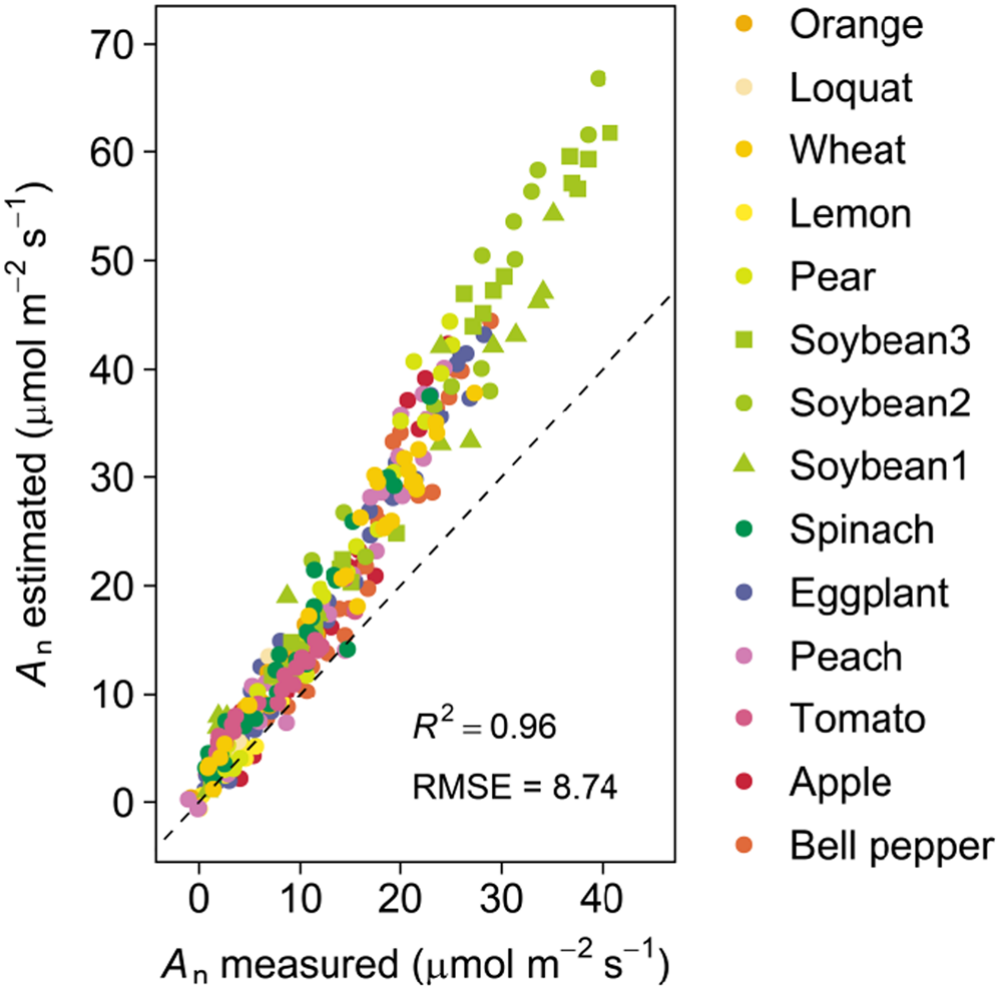
Relationship between estimated and measured leaf CO_2_ assimilation rate (*A*_n_) in 14 cultivars belonging to 12 species. *A*_n_ was estimated with the parameter *s* in Equation (3) fixing at the often‐used value of 0.42. The dashed line represents 1 to 1 line. [Color figure can be viewed at wileyonlinelibrary.com]

The biochemical photosynthesis model combined with measurements using a porometer and a fluorometer substantially reduced the environment‐ and species‐dependent biases in the estimation (Figure 4). However, *A*_n_ was significantly overestimated when the parameter *s* was fixed at 0.42 resulted from the often used values of *α* = 0.84 and *β* = 0.5 (Figure 4). Furthermore, the degree of overestimation increased as *A*_n_ increased (Figure 4), indicating an intensity‐dependent change in *s*.

**Figure 5:**
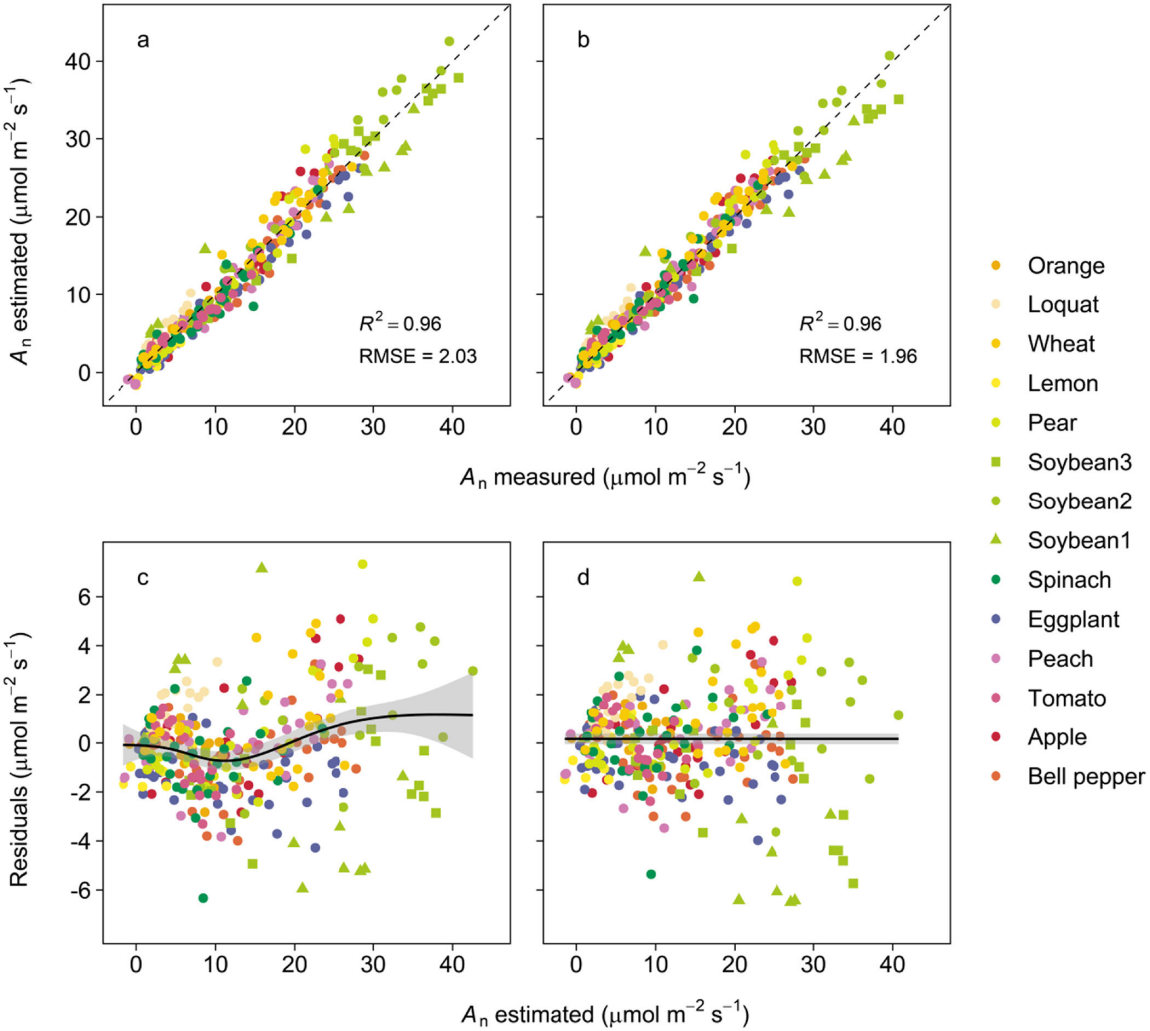
Relationship between estimated and measured leaf CO_2_ assimilation rate (*A*_n_) in 14 cultivars belonging to 12 species. *A*_n_ was estimated using parameter *s* in Equation (3) calibrating under the assumption that (a and c) *s* is constant and (b and d) *s* varies with quantum yield of photochemistry in PSII (Φ_PSII_) × photosynthetic photon flux density (PPFD). In (a and b), the results of cross‐validation are shown, and the dashed lines represent 1 to 1 line. In (c and d), residuals of *A*_n_ and their biased lines fitted using a generalized additive model are shown. [Color figure can be viewed at wileyonlinelibrary.com]

The calibration of *s* reduced the overestimation of *A*_n_ and reproduced the variations in the measured *A*_n_ across diverse environments and species (Figure 5a–d). Calibration using the constant *s* resulted in good estimates of *A*_n_ (Figure 5a) although negative and positive error biases were observed, ranging from 5 to 15 μmol m^−2^ s^−1^ and exceeding 20 μmol m^−2^ s^−1^, respectively (Figure 5c). Calibration using the variable *s* as a function of Φ_PSII_PPFD reduced the biases compared with the constant *s* approach, providing more reasonable *A*_n_ estimates (Figure 5b,d). For both constant and variable *s*, individual calibration for each species further improved estimation accuracy (Supporting Information S1: Figures S4 and S5), indicating species‐ and cultivar‐dependent *s* (Supporting Information S1: Table S1).

**Figure 6:**
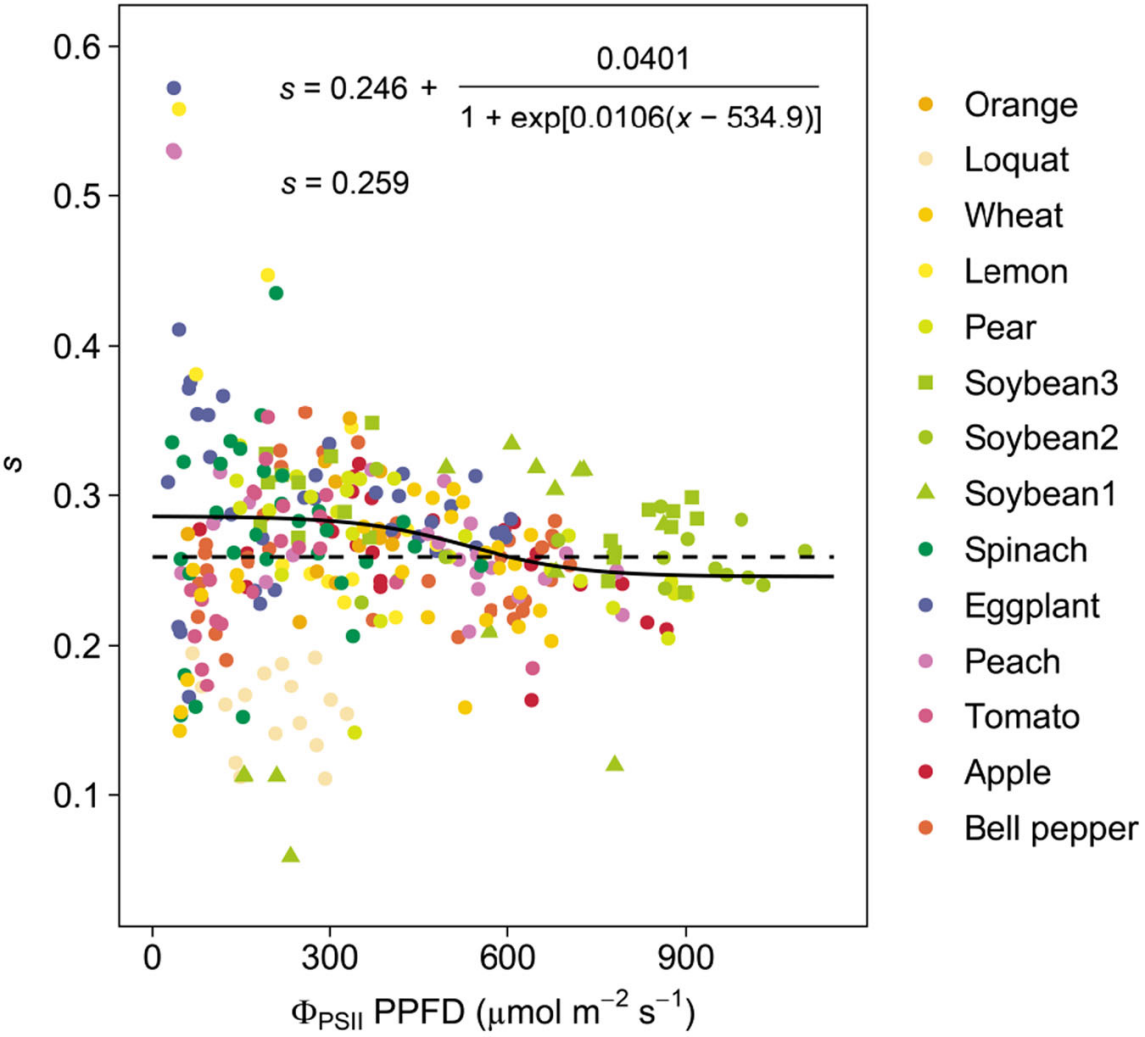
Responses of calibrated parameter *s* in Equation (3) to quantum yield of photochemistry in PSII (Φ_PSII_) × photosynthetic photon flux density (PPFD). *s* was calculated from Equations (2) and (3) by substituting the measured values of *A*_n_, *C*_i_, Φ_PSII_, and PPFD and the estimated values of Γ* and *R*_d_ from Equations (6)–(8). The regression lines and fitted equations under the assumption that *s* is constant (dashed line) and that *s* varies with Φ_PSII_PPFD (solid line) are also shown. [Color figure can be viewed at wileyonlinelibrary.com]

The calibrated value of constant *s* for all species was 0.259, which was 38% lower than the often‐used value (dashed line in Figure 6). The variable *s* ranged from 0.286 to 0.246 as Φ_PSII_PPFD increased (solid line in Figure 6), 32%–41% lower than the often‐used value. The large variations in individual *s* values at low Φ_PSII_PPFD values, shown in Figure 6, were due to the uncertainty in *R*_d_ when calculating *s*, rather than the actual variations in *s*. Equations (2) and (3) show the relative influence of *R*_d_ on *s*, where it becomes more significant when ΦPSII × PPFD is low.

## 4 Discussion

### 4.1 Accuracy of Estimating Leaf CO_2_ Assimilation Using the Porometer‐Fluorometer Method

The porometer‐fluorometer method measures both *g*_s_ and Φ_PSII_, enabling the consideration of both stomatal and non‐stomatal photosynthesis limitations. Consequently, this method improves the accuracy of *A*_n_ estimates compared to conventional empirical models that use only one of these variables (Figure 5 and Supporting Information S1: Figure S3). Additionally, the parameters of these empirical models show substantial species‐specific variations and vary with growth environments, even within the same species. This increases errors in *A*_n_ estimates when the empirical models are applied across a wide range of species and environmental conditions. In contrast, the calibrated parameter *s* in the porometer‐fluorometer method shows significantly smaller variations than the parameters of the empirical models (Supporting Information S1: Figure S3, Table S1), thereby reducing uncertainties in estimating *A*_n_ under varying species and environmental conditions. In summary, the porometer‐fluorometer method enables accurate *A*_n_ estimation in diverse C_3_ plants compared to conventional empirical approaches, provided appropriate calibration is performed.

In this study, the porometer‐fluorometer method was validated under relatively stable environmental conditions. However, its accuracy under fluctuating environments, particularly fluctuating light conditions that cause highly variable and delayed photosynthetic response (i.e., photosynthetic induction), in addition to fluctuating VPD conditions that may cause decline in *g*_s_ and *A*_n_ (Inoue et al. 2021), remains unclear. Short measurement intervals of variables can reduce the estimation error of *A*_n_ due to photosynthetic induction even when assuming a steady‐state (Murakami and Jishi 2022). Using a “Manual Mode” of the LI‐600 porometer, which allows continuous recording at intervals of 1–2 s while keeping the leaf clamped, may address this requirement. However, further investigation is required to determine the acceptable measurement interval and whether such an interval is feasible with a porometer and fluorometer.

### 4.2 Remaining Uncertainties in the Calibrated Parameter *s* and Possible Ideas to Reduce Them

The porometer‐fluorometer method effectively estimates *A*_n_ across diverse species, cultivars, and environments; however, uncertainties in the calibrated parameter *s*, require further investigation for more accurate estimation of *A*_n_. Previous studies indicate that *s* may be consistently lower than the often‐used value of 0.42. Loreto et al. (2009) found that *s* was 35–45% lower under artificial light with a high blue‐to‐red ratio, whereas McClain and Sharkey (2020) observed a 27%–31% decrease in *s*, likely due to blue light absorption by nonphotosynthetic pigments, such as carotenoids. Yin et al. (2009) estimated *s* to be 5%–20% lower than the often‐used values, suggesting alternative electron transport pathways such as cyclic electron flow. Another source of lower *s* values is Φ_PSII_ overvaluations caused by mesophyll layer heterogeneity within the leaf. Evans (2009) demonstrated that mesophyll layers have different Φ_PSII_ because of different photochemical properties and amount of absorbed light in each layer, causing overvaluations when measuring Φ_PSII_ on one side of leaf surface. These overvaluations increased under actinic light with high blue‐to‐red ratios, likely owing to high blue absorption in the top layers of the leaf and concomitant large heterogeneity (Evans et al. 2017). Moreover, Evans (2009) reported that heterogeneity increased with increasing light intensity, and these intensity‐dependent changes were large under higher blue‐to‐red light ratios (Evans et al. 2017). Natural sunlight contains higher blue fractions (approximately 50%: Kotilainen et al. 2020) than commonly used artificial lights (such as 10% blue). Therefore, the low values and intensity‐dependent changes in *s* observed in this study were likely driven by non‐photosynthetic light absorption and/or mesophyll layer heterogeneity, with alternative electron transport pathways playing a small role. Additionally, the present study found that woody plants, which tend to have thicker leaves and thus greater mesophyll layer heterogeneity than herbaceous species (Borsuk and Brodersen 2019), exhibited more pronounced light intensity‐dependent changes in *s* (Supporting Information S1: Figure S4), supporting the hypothesis that overvaluations of Φ_PSII_ owing to mesophyll layer heterogeneity affected variability in *s*. However, this study could not independently assess the impact of each factor, highlighting the need for further investigation under sunlight conditions.

Sunlight spectrum changes diurnally and seasonally with change in solar elevation angles and atmospheric conditions. Therefore, it is reasonable to assume that the value of *s* also varies over time. In the present study, however, even a constant value of *s* enabled accurate estimation of *A*_n_ although validation was performed across a range of seasons and times of day, suggesting a limited effect of temporal variation in *s* on *A*_n_ estimates. This is likely because diurnal and seasonal variations of blue‐to‐red ratios of sunlight are generally modest under many conditions (typically approximately 45%–55% at solar elevation angles > 20°: Kotilainen et al. 2020). Nevertheless, under conditions where the blue‐to‐red light ratio changes dramatically (at solar elevation angles < 20°), temporal variability in *s* may become more significant.

Uncertainties in *s* described above could be reduced using some approaches. The effects of non‐photosynthetic light absorption can be corrected using relative blue efficiency (ratio of quantum yield for blue and red light) (McClain and Sharkey 2020). Directly measuring mesophyll layer heterogeneity in the field remains challenging; however, a possible approach is to estimate it empirically using the leaf adaxial/abaxial chlorophyll fluorescence ratio, which is related to the intra‐leaf light absorption gradient (Mand et al. 2013). This ratio can be easily obtained using a fluorometer by turning the leaf over without additional instruments. Additionally, machine learning methods based on leaf hyperspectral reflectance can detect chloroplast movements (Hermanowicz and Łabuz 2024), potentially correcting for mesophyll heterogeneity effects varying with light conditions.

Uncertainties in conventional model parameters also affect the value of *s* in *A*_n_ estimation. Typically, *α* is assumed to be approximately 0.85, and this assumption is reasonable for *A*_n_ estimates in most healthy leaves (Supporting Information S1: Figure S6). However, *α* should be carefully determined for senescent or drought‐stressed leaves, as it may be significantly lower (Gates 1980; Ehleringer 1980). *α* values depend on leaf chlorophyll content (Gabrielsen 1948) and thus, can be individually estimated from spectral indices to retrieve leaf chlorophyll content (e.g., the present study used NDVIgreen for estimating *α* in 12 species; Supporting Information S1: Figure S6). Often, *β* is assumed to be 0.5, but its value varies under far‐red light conditions (Wientjes et al. 2017; Murakami et al. 2018). However, individual determination of *β* is challenging under field conditions. Although Γ* value is often taken from a literature source, it generally varies among species (Hermida‐Carrera et al. 2016). The present study predicted that an extremely overestimated or underestimated Γ* value would increase the error in *A*_n_ estimate, although a dire error in *A*_n_ could be avoided (Supporting Information S1: Figure S7). Individual determination of Γ* can provide more reasonable *A*_n_ estimates, but it remains challenging, as well as in the case of *β*. In the present study, *g*_m_ was assumed to be infinite, and the effect of *g*_m_ on *A*_n_ was partially included in the calibrated values of *s*. This assumption is useful when *g*_m_ is independently high or comparable to or higher than *g*_sc_, as is likely the case for most of the data in this study (Supporting Information S1: Figure S8). However, assuming infinite *g*_m_ does not hold under certain conditions. When *g*_m_ coordinates with *g*_sc_, estimation error in *A*_n_ increases when an absolute value of *g*_m_ is low and its value is lower than that of *g*_sc_ (Supporting Information S1: Figure S8). These errors cannot be fully corrected using only a single parameter *s* (Supporting Information S1: Figure S8). Therefore, in leaves under such extreme conditions (e.g., severe drought), individual determination or calibration of *g*_m_ is recommended for a more accurate estimation of *A*_n_ (Niinemets et al. 2009). Uncertainties in *R*_d_ are more problematic than those in other parameters because *R*_d_ is the intercept in Equation (2), and parameter *s*, which is the slope in Equation (2), cannot fully calibrate the effect of *R*_d_. In particular, *R*_d_ should be carefully determined at a low *A*_n_, where the relative contribution of *R*_d_ is large (Supporting Information S1: Figure S9). *g*_m_ and *R*_d_ can be simultaneously estimated using the non‐rectangular hyperbolic model combined with measurements of gas exchange and chlorophyll fluorescence under photorespiratory conditions (i.e., under ambient air conditions) (Yin and Struik 2009; Yin and Amthor 2024), although this method requires step changes in CO_2_ and PPFD.

Despite these uncertainties, the proposed method reproduced *A*_n_ variations with a common parameter set across species. This may be due to reduced parameter variations through coordination within the photosynthetic system between electron transport and carbon metabolism. Assuming that the energy distribution in photosystems and cyclic and pseudo‐cyclic electron transports are coordinated with linear electron transport to fulfill carbon metabolism demands (Allen 2003; Johnson and Berry 2021), their effects (i.e., *β*, *f*_pseudo_, *f*_cyc_, and resultant *s* in Equation [3]) are functions of Φ_PSII_, as modeled in this study. Thus, variations in *β*, *f*_pseudo_, and *f*_cyc_ are partly reflected in the measured Φ_PSII_, leading to smaller variations in the calibrated parameter *s* compared to scenarios without coordination. However, such coordination is nonlinear, and there are limits to imposing all uncertainties on one parameter. Ideally, these parameters discussed in this section should be determined individually to reduce the uncertainties in *s*. However, individual estimations are currently time‐consuming or technically difficult under field conditions, which conflicts with the goal of high‐throughput estimation of *A*_n_. The proposed method, which incorporates some uncertainties into the parameter *s*, offers a temporary solution that balances the accuracy, applicability, and efficiency in estimating *A*_n_ in the field.

### 4.3 Advantages and Disadvantages of the Porometer‐Fluorometer Method

#### 4.3.1 Versus Gas Exchange Measurements

The compact, lightweight design of the porometer with the chlorophyll fluorometer enables on‐the‐go measurements in the field, and its fast stabilization time allows researchers to obtain high spatiotemporal resolution data on *A*_n_. This setup is more advantageous for high‐throughput field phenotyping of photosynthesis than gas exchange measurements. While using multiple gas exchange systems can also perform high‐throughput phenotyping, only a limited number of researchers can afford this approach because of its cost and the manpower required. The porometer‐fluorometer method offers a cost‐effective and labor‐efficient alternative, making it accessible to a broad range of researchers. Moreover, the simple process of clamping leaves in addition to the compact design may enable the automatic estimation of *A*_n_ using techniques for harvesting robots developed in greenhouse agriculture (Zhao et al. 2016). For example, a robot that can automatically detect and clamp leaves with a porometer and chlorophyll fluorometer mounted on its arm can be expected. This further reduces labor costs and increases the efficiency of collecting data of *A*_n_.

The porometer‐fluorometer method requires empirical calibration due to uncertainties in plant physiological responses, whereas gas exchange measurements are primarily based on the physical process of leaf gas exchange and can independently measure *A*_n_ from the physiological mechanisms. Therefore, the accuracy of *A*_n_ evaluation in the porometer‐fluorometer method is lower than that of gas exchange measurements, and for precise *A*_n_ evaluation, the conventional gas exchange system remains the gold standard. The porometer‐fluorometer method may be suitable for identifying leaves and cultivars with relatively high *A*_n_ for screening (such as breeding programs in agriculture) but less so for detailed studies of specific photosynthetic processes where accuracy is paramount.

#### 4.3.2 Versus Proximal and Remote Sensing Techniques

The porometer‐fluorometer method provides contact measurement of Φ_PSII_ and *g*_s_ to estimate *A*_n_. In contrast, recent advances in light‐induce fluorescence transient (LIFT) method (Kolber et al. 1998; Keller et al. 2019) and sun‐induced chlorophyll fluorescence (SIF) measurement (Porcar‐Castell et al. 2021) have enabled proximal and remote sensing of Φ_PSII_ (or related variables) across various scales. Additionally, leaf temperature measurements combined with energy balance analysis enable proximal and remote sensing of *g*_s_ (Jones 1999; Vialet‐Chabrand and Lawson 2019). These techniques can be also combined with a biochemical photosynthesis model to estimate *A*_n_ (Kimura et al. 2024), as performed in this study. Unlike the porometer‐fluorometer method, these techniques not only allow for the noncontact estimation of *A*_n_ but also enable the instant detection of spatial distributions of *A*_n_ when combined with imaging techniques. Therefore, throughput of the porometer‐fluorometer method may be lower than that of proximal and remote sensing approaches. However, instruments simultaneously detecting Φ_PSII_ and *g*_s_ for proximal and remote sensing have not yet been commercialized, and thus the porometer‐fluorometer method is currently the most accessible for most researchers.

The porometer‐fluorometer method measures *g*_s_ and Φ_PSII_ directly, reducing uncertainties in *A*_n_ estimates compared with relying on empirical estimates of *g*_s_ and Φ_PSII_. In contrast, proximal and remote sensing approaches require additional models to estimate *J* from SIF (Gu et al. 2019; Han et al. 2022) and supplementary measurements using reference materials to estimate *g*_s_ from thermal imaging (Jones 1999; Vialet‐Chabrand and Lawson 2019), increasing uncertainties in *A*_n_ estimates. Therefore, *A*_n_ estimate accuracy in the porometer‐fluorometer method is generally higher than that when using proximal and remote sensing approaches.

#### 4.3.3 Combination With the Conventional Methods

Disadvantages of the porometer‐fluorometer method can be compensated in combination with gas exchange measurements and proximal and remote sensing techniques. The porometer‐fluorometer method can complement gas exchange systems, offering high‐throughput sampling across a large field with precise calibration of small representative samples. Moreover, this method can be used to calibrate *g*_s_ and Φ_PSII_ estimated from proximal and remote sensing, further increasing the throughput of *A*_n_ estimates with maintained accuracy. Overall, the porometer‐fluorometer method is not necessarily a replacement for existing methods and is valuable when used in combination with other methods.

## Conflicts of Interest

The authors declare no conflicts of interest.

## Supporting information


**Supporting_Information_S1.docx**: Microsoft Word file including supplemental figures, tables, and notes.

**Supporting_Information_S2_Dataset.xlsx**: Microsoft Excel sheet including dataset used in this study and formulae to calculate *A*_n_.

**Supporting_Information_S3_R_program.R**: R program to calibrate the parameter s and perform cross‐validation.

## Data Availability

The data used to reproduce the results of this study are available in Supporting Information S2 and S3. Supporting Information S2: Microsoft Excel sheet including data and formulae for calculating An. Supporting Information S3: R program for calibrating the parameter s and performing cross‐validation, and Supporting Information S2 can be read into this program as a template. Directions for their usage are described in the files.

## References

[pce70006-bib-0001] Allen, J. F. 2003. “Cyclic, Pseudocyclic and Noncyclic Photophosphorylation: New Links in the Chain.” Trends in Plant Science 8: 15–19. 12523995 10.1016/s1360-1385(02)00006-7

[pce70006-bib-0002] Borsuk, A. M. , and C. R. Brodersen . 2019. “The Spatial Distribution of Chlorophyll in Leaves.” Plant Physiology 180: 1406–1417. 30944156 10.1104/pp.19.00094 PMC6752913

[pce70006-bib-0003] Chow, W. S. , A. Melis , and J. M. Anderson . 1990. “Adjustments of Photosystem Stoichiometry in Chloroplasts Improve the Quantum Efficiency of Photosynthesis.” Proceedings of the National Academy of Sciences 87: 7502–7506. 10.1073/pnas.87.19.7502 PMC54775 11607105

[pce70006-bib-0004] Ehleringer, J. 1980. “Leaf Morphology and Reflectance in Relation to Water and Temperature Stress.” In Adaptations of Plants to Water and High Temperature Stress, edited by N. C. Turner and P. J. Kramer , 295–308. Wiley.

[pce70006-bib-0005] Evans, J. R. 2009. “Potential Errors in Electron Transport Rates Calculated From Chlorophyll Fluorescence as Revealed by a Multilayer Leaf Model.” Plant and Cell Physiology 50: 698–706. 19282373 10.1093/pcp/pcp041

[pce70006-bib-0006] Evans, J. R. , P. B. Morgan , and S. Von Caemmerer . 2017. “Light Quality Affects Chloroplast Electron Transport Rates Estimated From Chl Fluorescence Measurements.” Plant and Cell Physiology 58: 1652–1660. 29016964 10.1093/pcp/pcx103

[pce70006-bib-0007] Fang, L. , X. Yin , P. E. L. van der Putten , P. Martre , and P. C. Struik . 2022. “Drought Exerts a Greater Influence Than Growth Temperature on the Temperature Response of Leaf Day Respiration in Wheat (*Triticum aestivum*).” Plant, Cell and Environment 45: 2062–2077. 10.1111/pce.14324 PMC9324871 35357701

[pce70006-bib-0008] Farquhar, G. D. , S. von Caemmerer , and J. A. Berry . 1980. “A Biochemical Model of Photosynthetic CO_2_ Assimilation in Leaves of C_3_ Species.” Planta 149: 78–90. 24306196 10.1007/BF00386231

[pce70006-bib-0009] Gabrielsen, E. K. 1948. “Effects of Different Chlorophyll Concentrations on Photosynthesis in Foliage Leaves.” Physiologia Plantarum 1: 5–37.

[pce70006-bib-0010] Galmés, J. , C. Hermida‐Carrera , L. Laanisto , and Ü. Niinemets . 2016. “A Compendium of Temperature Responses of Rubisco Kinetic Traits: Variability Among and Within Photosynthetic Groups and Impacts on Photosynthesis Modeling.” Journal of Experimental Botany 67: 5067–5091. 27406782 10.1093/jxb/erw267 PMC5014154

[pce70006-bib-0011] Gates, D. M. 1980. Biophysical Ecology, 215–238. Springer‐Verlag.

[pce70006-bib-0012] Genty, B. , J. M. Briantais , and N. R. Baker . 1989. “The Relationship Between the Quantum Yield of Photosynthetic Electron Transport and Quenching of Chlorophyll Fluorescence.” Biochimica et Biophysica Acta (BBA) ‐ General Subjects 990: 87–92.

[pce70006-bib-0013] Gu, L. , J. Han , J. D. Wood , C. Y. Y. Chang , and Y. Sun . 2019. “Sun‐Induced Chl Fluorescence and its Importance for Biophysical Modeling of Photosynthesis Based on Light Reactions.” New Phytologist 223: 1179–1191. 30883811 10.1111/nph.15796

[pce70006-bib-0014] Han, J. , C. Y. Y. Chang , L. Gu , et al. 2022. “The Physiological Basis for Estimating Photosynthesis From Chl a Fluorescence.” New Phytologist 234: 1206–1219. 35181903 10.1111/nph.18045

[pce70006-bib-0015] Harley, P. C. , F. Loreto , G. Di Marco , and T. D. Sharkey . 1992. “Theoretical Considerations When Estimating the Mesophyll Conductance to CO_2_ Flux by Analysis of the Response of Photosynthesis to CO_2_ .” Plant Physiology 98: 1429–1436. 16668811 10.1104/pp.98.4.1429 PMC1080368

[pce70006-bib-0016] He, D. , and G. E. Edwards . 1996. “Evaluation of the Potential to Measure Photosynthetic Rates in C_3_ Plants (*Flaveria pringlei* and *Oryza sativa*) by Combining Chlorophyll Fluorescence Analysis and a Stomatal Conductance Model.” Plant, Cell and Environment 19: 1272–1280.

[pce70006-bib-0017] Hermanowicz, P. , and J. Łabuz . 2024. “Hyperspectral Imaging for Chloroplast Movement Detection.” Journal of Experimental Botany 76, no. 3: 882–898. 10.1093/jxb/erae407 PMC11805589 39329458

[pce70006-bib-0018] Hermida‐Carrera, C. , M. V. Kapralov , and J. Galmés . 2016. “Rubisco Catalytic Properties and Temperature Response in Crops.” Plant Physiology 171: 2549–2561. 27329223 10.1104/pp.16.01846 PMC4972260

[pce70006-bib-0019] Inoue, T. , M. Sunaga , M. Ito , et al. 2021. “Minimizing VPD Fluctuations Maintains Higher Stomatal Conductance and Photosynthesis, Resulting in Improvement of Plant Growth in Lettuce.” Frontiers in Plant Science 12: 646144. 33868345 10.3389/fpls.2021.646144 PMC8049605

[pce70006-bib-0020] Johnson, J. E. , and J. A. Berry . 2021. “The Role of Cytochrome b6f in the Control of Steady‐State Photosynthesis: A Conceptual and Quantitative Model.” Photosynthesis Research 148: 101–136. 33999328 10.1007/s11120-021-00840-4 PMC8292351

[pce70006-bib-0021] Jones, H. G. 1999. “Use of Thermography for Quantitative Studies of Spatial and Temporal Variation of Stomatal Conductance over Leaf Surfaces.” Plant, Cell and Environment 22: 1043–1055.

[pce70006-bib-0022] Keller, B. , I. Vass , S. Matsubara , et al. 2019. “Maximum Fluorescence and Electron Transport Kinetics Determined by Light‐Induced Fluorescence Transients (LIFT) for Photosynthesis Phenotyping.” Photosynthesis Research 140: 221–233. 30357678 10.1007/s11120-018-0594-9 PMC6548062

[pce70006-bib-0023] Kimura, K. , E. Kumagai , E. Fushimi , and A. Maruyama . 2024. “Alternative Method for Determining Leaf CO2 Assimilation Without Gas Exchange Measurements: Performance, Comparison and Sensitivity Analysis.” Plant, Cell and Environment 47: 992–1002. 10.1111/pce.14780 38098202

[pce70006-bib-0024] Kitao, M. , Y. Yasuda , E. Kodani , et al. 2021. “Integration of Electron Flow Partitioning Improves Estimation of Photosynthetic Rate under Various Environmental Conditions Based on Chlorophyll Fluorescence.” Remote Sensing of Environment 254: 112273.

[pce70006-bib-0025] Kolber, Z. S. , O. Prášil , and P. G. Falkowski . 1998. “Measurements of Variable Chlorophyll Fluorescence Using Fast Repetition Rate Techniques: Defining Methodology and Experimental Protocols.” Biochimica et Biophysica Acta (BBA) ‐ Bioenergetics 1367: 88–106. 9784616 10.1016/s0005-2728(98)00135-2

[pce70006-bib-0026] Kotilainen, T. , P. Aphalo , C. Brelsford , et al. 2020. “Patterns in the Spectral Composition of Sunlight and Biologically Meaningful Spectral Photon Ratios as Affected by Atmospheric Factors.” Agricultural and Forest Meteorology 291: 108041.

[pce70006-bib-0027] Laisk, A. , and F. Loreto . 1996. “Determining Photosynthetic Parameters From Leaf CO2 Exchange and Chlorophyll Fluorescence (Ribulose‐1,5‐Bisphosphate Carboxylase/Oxygenase Specificity Factor, Dark Respiration in the Light, Excitation Distribution Between Photosystems, Alternative Electron Transport Rate, and Mesophyll Diffusion Resistance.” Plant Physiology 110: 903–912. 12226229 10.1104/pp.110.3.903 PMC157790

[pce70006-bib-0028] Leuning, R. 1995. “A Critical Appraisal of Combine Stomatal Model C3 Plants.” Plant, Cell and Environment 18: 339–355.

[pce70006-bib-0029] Loreto, F. , T. Tsonev , and M. Centritto . 2009. “The Impact of Blue Light on Leaf Mesophyll Conductance.” Journal of Experimental Botany 60: 2283–2290. 19395388 10.1093/jxb/erp112

[pce70006-bib-0030] Loriaux, S. D. , T. J. Avenson , J. M. Welles , et al. 2013. “Closing in on Maximum Yield of Chlorophyll Fluorescence Using a Single Multiphase Flash of Sub‐Saturating Intensity.” Plant, Cell and Environment 36: 1755–1770. 10.1111/pce.12115 23586649

[pce70006-bib-0031] Mand, P. , L. Hallik , J. Penuelas , and O. Kull . 2013. “Electron Transport Efficiency At Opposite Leaf Sides: Effect of Vertical Distribution of Leaf Angle, Structure, Chlorophyll Content and Species in a Forest Canopy.” Tree Physiology 33: 202–210. 23185067 10.1093/treephys/tps112

[pce70006-bib-0032] McClain, A. M. , and T. D. Sharkey . 2020. “Building a Better Equation for Electron Transport Estimated From Chl Fluorescence.” New Phytologist 225: 604–608. 31605374 10.1111/nph.16255 PMC7660523

[pce70006-bib-0033] Murakami, K. , and T. Jishi . 2022. “Appropriate Time Interval of PPFD Measurement to Estimate Daily Photosynthetic Gain.” Functional Plant Biology 49: 452–462. 33549153 10.1071/FP20323

[pce70006-bib-0034] Murakami, K. , R. Matsuda , and K. Fujiwara . 2018. “Quantification of Excitation Energy Distribution Between Photosystems Based on a Mechanistic Model of Photosynthetic Electron Transport.” Plant, Cell and Environment 41: 148–159. 10.1111/pce.12986 28548208

[pce70006-bib-0035] Niinemets, Ü. , A. Díaz‐Espejo , J. Flexas , J. Galmés , and C. R. Warren . 2009. “Importance of Mesophyll Diffusion Conductance in Estimation of Plant Photosynthesis in the Field.” Journal of Experimental Botany 60: 2271–2282. 19305021 10.1093/jxb/erp063

[pce70006-bib-0036] Porcar‐Castell, A. , Z. Malenovský , T. Magney , et al. 2021. “Chlorophyll a Fluorescence Illuminates a Path Connecting Plant Molecular Biology to Earth‐System Science.” Nature Plants 7: 998–1009. 34373605 10.1038/s41477-021-00980-4

[pce70006-bib-0037] van der Putten, P. E. L. , X. Yin , and P. C. Struik . 2018. “Calibration Matters: On the Procedure of Using the Chlorophyll Fluorescence Method to Estimate Mesophyll Conductance.” Journal of Plant Physiology 220: 167–172. 29190520 10.1016/j.jplph.2017.11.009

[pce70006-bib-0038] Saathoff, A. J. , and J. Welles . 2021. “Gas Exchange Measurements in the Unsteady State.” Plant, Cell and Environment 44: 3509–3523. 10.1111/pce.14178 PMC9292621 34480484

[pce70006-bib-0039] Sharkey, T. D. 1985. “Photosynthesis in Intact Leaves of C3 Plants: Physics, Physiology and Rate Limitations.” Botanical Review 51: 53–105.

[pce70006-bib-0040] Simkin, A. J. , P. E. López‐Calcagno , and C. A. Raines . 2019. “Feeding the World: Improving Photosynthetic Efficiency for Sustainable Crop Production.” Journal of Experimental Botany 70: 1119–1140. 30772919 10.1093/jxb/ery445 PMC6395887

[pce70006-bib-0041] Vialet‐Chabrand, S. , and T. Lawson . 2019. “Dynamic Leaf Energy Balance: Deriving Stomatal Conductance From Thermal Imaging in a Dynamic Environment.” Journal of Experimental Botany 70: 2839–2855. 30793211 10.1093/jxb/erz068 PMC6506762

[pce70006-bib-0042] Wientjes, E. , J. Philippi , J. W. Borst , and H. van Amerongen . 2017. “Imaging the Photosystem I/Photosystem II Chlorophyll Ratio Inside the Leaf.” Biochimica et Biophysica Acta (BBA)–Bioenergetics 1858: 259–265. 28095301 10.1016/j.bbabio.2017.01.008

[pce70006-bib-0043] Yin, X. , and J. S. Amthor . 2024. “Estimating Leaf Day Respiration From Conventional Gas Exchange Measurements.” New Phytologist 241: 52–58. 37858976 10.1111/nph.19330

[pce70006-bib-0044] Yin, X. , and P. C. Struik . 2009. “Theoretical Reconsiderations When Estimating the Mesophyll Conductance to CO_2_ Diffusion In Leaves of C_3_ Plants by Analysis of Combined Gas Exchange and Chlorophyll Fluorescence Measurements.” Plant, Cell and Environment 32: 1513–1524. 10.1111/j.1365-3040.2009.02016.x 19558403

[pce70006-bib-0045] Yin, X. , P. C. Struik , P. Romero , et al. 2009. “Using Combined Measurements of Gas Exchange and Chlorophyll Fluorescence to Estimate Parameters of a Biochemical C Photosynthesis Model: A Critical Appraisal and a New Integrated Approach Applied to Leaves in a Wheat (*Triticum aestivum*) Canopy.” Plant, Cell and Environment 32: 448–464. 10.1111/j.1365-3040.2009.01934.x 19183300

[pce70006-bib-0046] Zhao, Y. , L. Gong , Y. Huang , and C. Liu . 2016. “A Review of Key Techniques of Vision‐Based Control for Harvesting Robot.” Computers and Electronics in Agriculture 127: 311–323.

[pce70006-bib-0047] Zhu, X. G. , S. P. Long , and D. R. Ort . 2010. “Improving Photosynthetic Efficiency for Greater Yield.” Annual Review of Plant Biology 61: 235–261. 10.1146/annurev-arplant-042809-112206 20192734

