# Supplementary material for: Estimating Leaf CO2 Assimilation in C3 Plants Using a Handheld Porometer With Chlorophyll Fluorometer in Field Conditions
Source: Plant Cell Environ. 2025 Jun 18;48(10):7213–24. doi: 10.1111/pce.70006 (PMC12415414; doi:10.1111/pce.70006)
Supplement: Supplementary file 1 — Supporting_Information_S1.docx: Microsoft Word file including supplemental figures, tables, and notes. [file PCE-48-7213-s003.docx]

**Supporting Information S1**

**Estimating leaf CO_2_ assimilation in C_3_ plants using a handheld porometer with chlorophyll fluorometer in field conditions**

Kensuke Kimura, Erina Fushimi, Etsushi Kumagai, Koichi Nomura, Toshinori Matsunami, Shohei Konno, Atsushi Maruyama

The following Supporting Information is available for this article.

**Figure S1.** Variation of environmental and physiological variables measured in this study.

**Figure S2.** Effect of mismatch of the leaf total conductance (*g*_tc_) between LI-6800 and LI-600 on the estimate of leaf CO_2_ assimilation rate (*A*_n_).

**Figure S3.** *A*_n_ estimation accuracy using conventional empirical models.

**Figure S4.** Effect of individual calibration for woody and herbaceous species on *A*_n_ estimate using the porometer-fluorometer method.

**Figure S5.** Effect of individual calibration for each species and cultivar on *A*_n_ estimate using the porometer-fluorometer method.

**Figure S6.** Relationship between leaf absorptance to photosynthetic photon flux density (*α*) and green normalized difference vegetation index (NDVI_green_), and effect of independent *α* estimation on *A*_n_ estimate.

**Figure S7.** Effect of change in CO_2_ compensation point in the absence of day respiration (Γ^*^) on the calibrated parameter *s* and *A*_n_ estimate using the porometer-fluorometer method.

**Figure S8.** Effect of assuming infinite mesophyll conductance (*g*_m_) on the calibrated parameter *s* and *A*_n_ estimate using the porometer-fluorometer method when *g*_m_ is assumed to change with stomatal conductance (*g*_s_).

**Figure S9.** Effect of change in day respiration rate (*R*_d_) on the calibrated parameter *s* and *A*_n_ estimate using the porometer-fluorometer method.

**Table S1.** The fitted values of the parameter *s* for each species and cultivar.

**Note S1.** Analytical solution of the model for estimating *A*_n_.

**Note S2.** Growth conditions for plant materials.


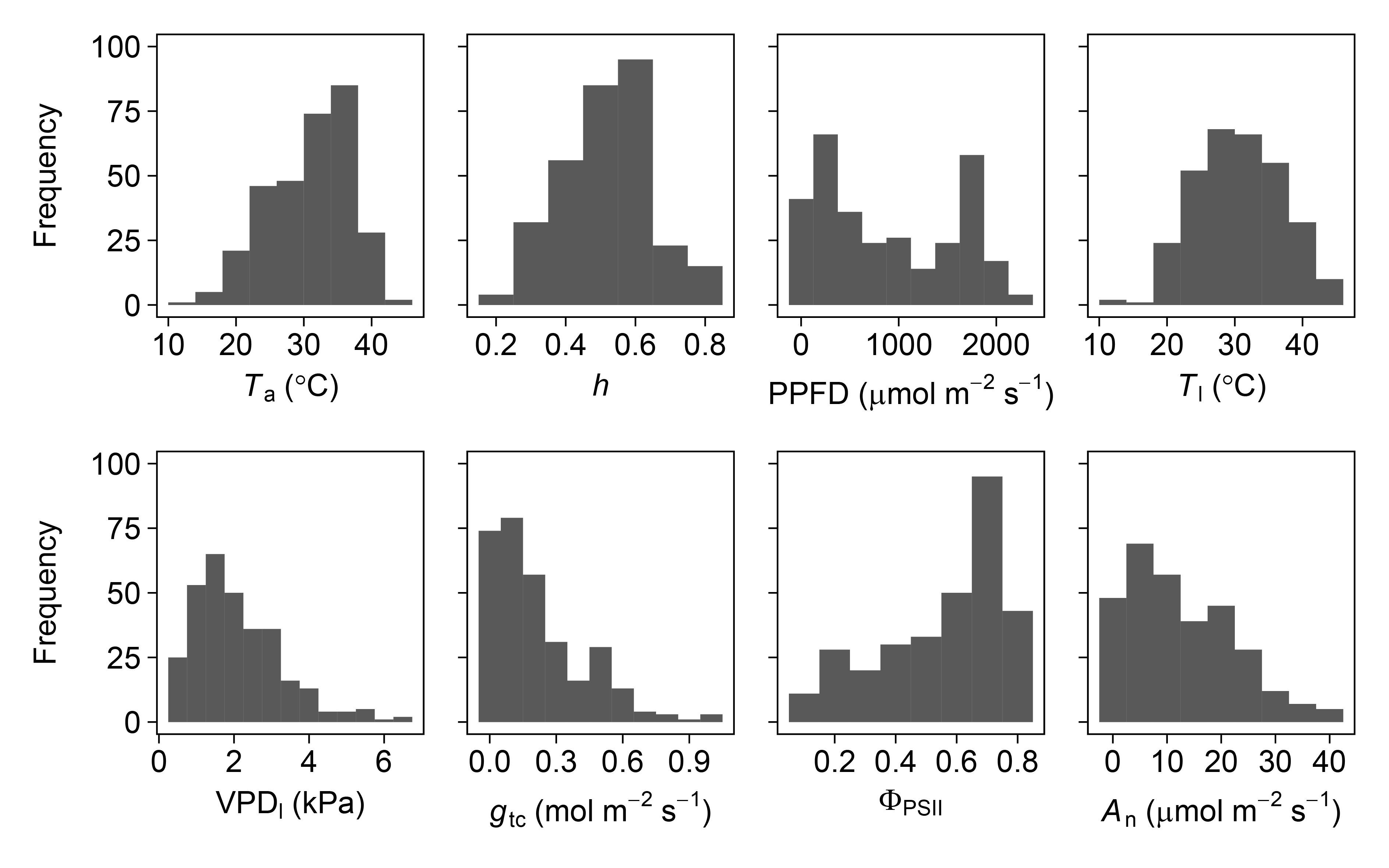


# Figure S1. Variations in air temperature (*T*_a_), relative humidity (*h*), photosynthetic photon flux density (PPFD), leaf temperature (*T*_l_), leaf vapor pressure deficit (VPD_l_), leaf total conductance for CO_2_ transfer (*g*_tc_), quantum yield of photochemistry in PSII (Φ_PSII_), and leaf CO_2_ assimilation rate (*A*_n_) during the experiment. *T*_a_, *h*, PPFD, *T*_l_, VPD_l_, *g*_tc_, and Φ_PSII_ were measured using the LI-600 porometer with fluorometer, and *A*_n_ was measured using the LI-6800 gas exchange system.


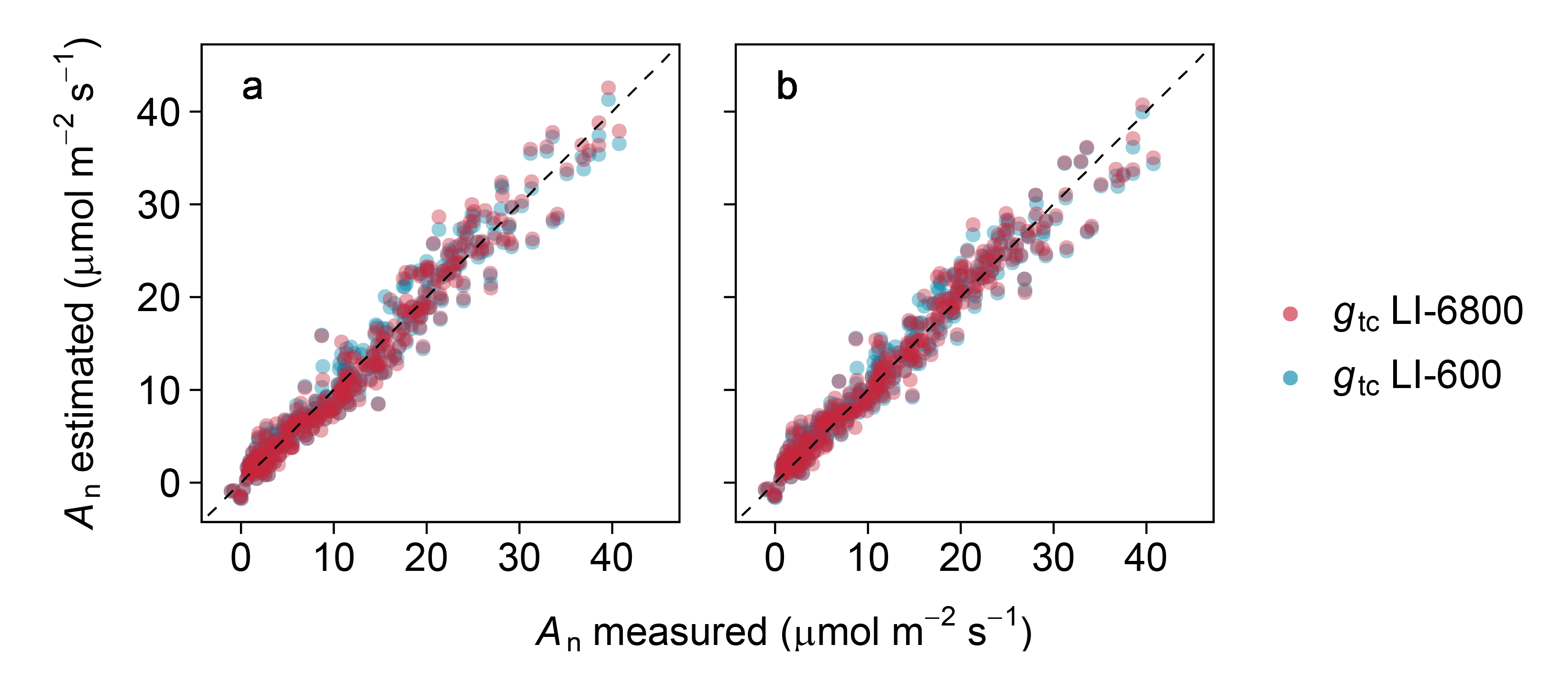


# Figure S2. Relationship between estimated and measured leaf CO_2_ assimilation rate (*A*_n_). *A*_n_ was estimated using the leaf total conductance for CO_2_ transfer (*g*_tc_) inferred from the LI-6800 gas exchange measurement system (red circle) and the LI-600 porometer with a chlorophyll fluorometer (blue circle). The results of cross-validation with (a) constant calibrated parameters *s* and (b) variable *s* as a function of quantum yield of photochemistry in PSII (Φ_PSII_) × photosynthetic photon flux density (PPFD) are shown.

Similar *A*_n_ values were obtained when either of the *g*_tc_ values inferred from both instruments were used. This result shows that the overestimation of *g*_tc_ in the LI-600 porometer at high *g*_tc_ values (Fig. 2f in the main manuscript) does not result in a dire error in the *A*_n_ estimate because *A*_n_ is primarily constrained by other factors when *g*_tc_ is significantly high.


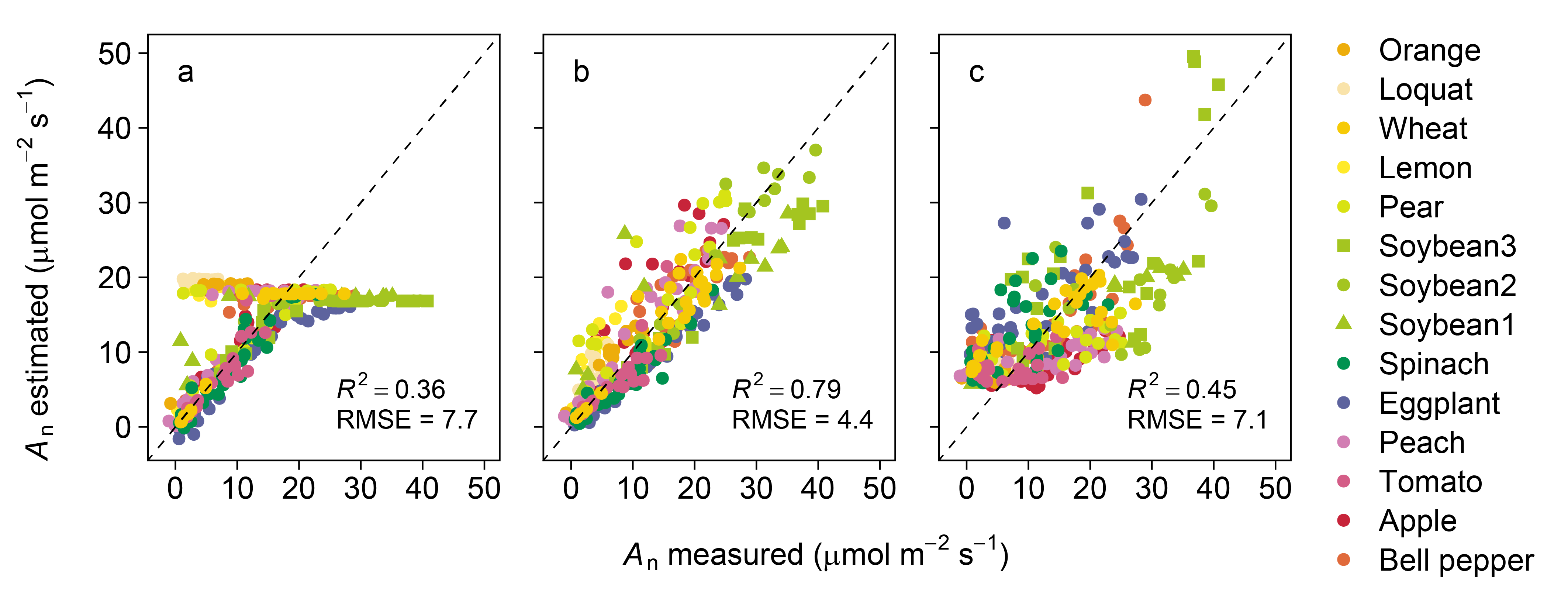


# Figure S3. Relationship between estimated and measured leaf CO_2_ assimilation rate (*A*_n_) in 14 cultivars among 12 species. *A*_n_ was estimated using conventional models: (a) the non-rectangular hyperbola equation as a function of photosynthetic photon flux density (PPFD), (b) the linear equation as a function of quantum yield of photochemistry in PSII (Φ_PSII_) × PPFD, and (c) deformation of the stomatal conductance (*g*_s_) model. The results of cross-validation are shown.

In Fig. S3a, *A*_n_ was estimated using the non-rectangular hyperbola equation as follows:

|  | $\text{​}\text{A}_{\text{n}}\text{ = }\frac{\text{φ}\text{PPFD}+ \text{A}_{\text{max}}-\sqrt{\left( \text{φ}\text{PPFD}+ \text{A}_{\text{max}} \right)^{2}-\text{4 }\text{φ}\text{PPFD}\text{A}_{\text{max}}\text{θ}}}{\text{2}\text{θ}}-\text{R}_{\text{n}}$ |  |
| --- | --- | --- |

where *φ* is the maximum quantum yield for CO_2_ uptake, *θ* is the convexity of the curve, *A*_max_ is the light-saturated rate of *A*_n_, and *R*_n_ is the dark respiration rate. *φ*, *θ*, *A*_max_, and *R*_n_ were calibrated by minimizing the root mean square error (RMSE) between the estimated and measured values of *A*_n_ using differential evolution with the DEoptim package in R. Although *A*_n_ was successfully estimated at low *A*_n_ values, it was not estimated at high *A*_n_ values due to species-specific variations in *A*_max_. The coefficient of variation (CV) of *A*_max_ fitted for each species was 58%.

In Fig. S3b, *A*_n_ was estimated using the linear equation as follows:

|  | $\text{​}\text{A}_{\text{n}}\text{ = }\text{a}\text{ }\text{Φ}_{\text{PSII}} \text{PPFD}-\text{b}$ |  |
| --- | --- | --- |

where a and b are the slope and intercept of the equation, respectively. a and b were calibrated by minimizing RMSE between the estimated and measured values of *A*_n_ using differential evolution with the DEoptim package in R. Although *A*_n_ was successfully estimated, its accuracy was lower than that estimated using the porometer-fluorometer method because the effects of stomatal limitation, which are imposed into the parameter a, cannot be accurately evaluated. The CV of the parameter a fitted for each species was 25%.

In Fig. S3c, *A*_n_ was estimated using deformation of the empirical stomatal conductance model (Ball et al., 1987) as follows:

|  | $\text{​}\text{A}_{\text{n}}\text{ = }\frac{\left( \text{g}_{\text{sw}}-\text{g}_{\text{0}} \right)\text{C}_{\text{a}}}{\text{g}_{\text{1}}\text{h}}$ |  |
| --- | --- | --- |

where *g*_sw_ is *g*_s_ for water vapor transfer, *C*_a_ is the atmospheric CO_2_ concentration, *h* is the relative humidity, and *g*_0_ and *g*_1_ are the empirical parameters. *g*_0_ and *g*_1_ were calibrated by minimizing RMSE between the estimated and measured values of *A*_n_ using differential evolution with the DEoptim package in R. The estimated values of *A*_n_ showed considerable dispersion compared to the measured values due to large variations in *g*_1_ among species. The CV of the parameter *g*_1_ fitted for each species was 80%.

In summary, the individual variables of PPFD, Φ_PSII_, and *g*_s_ alone provided less accurate *A*_n_ estimates across different environments and species, compared to the porometer-fluorometer method.


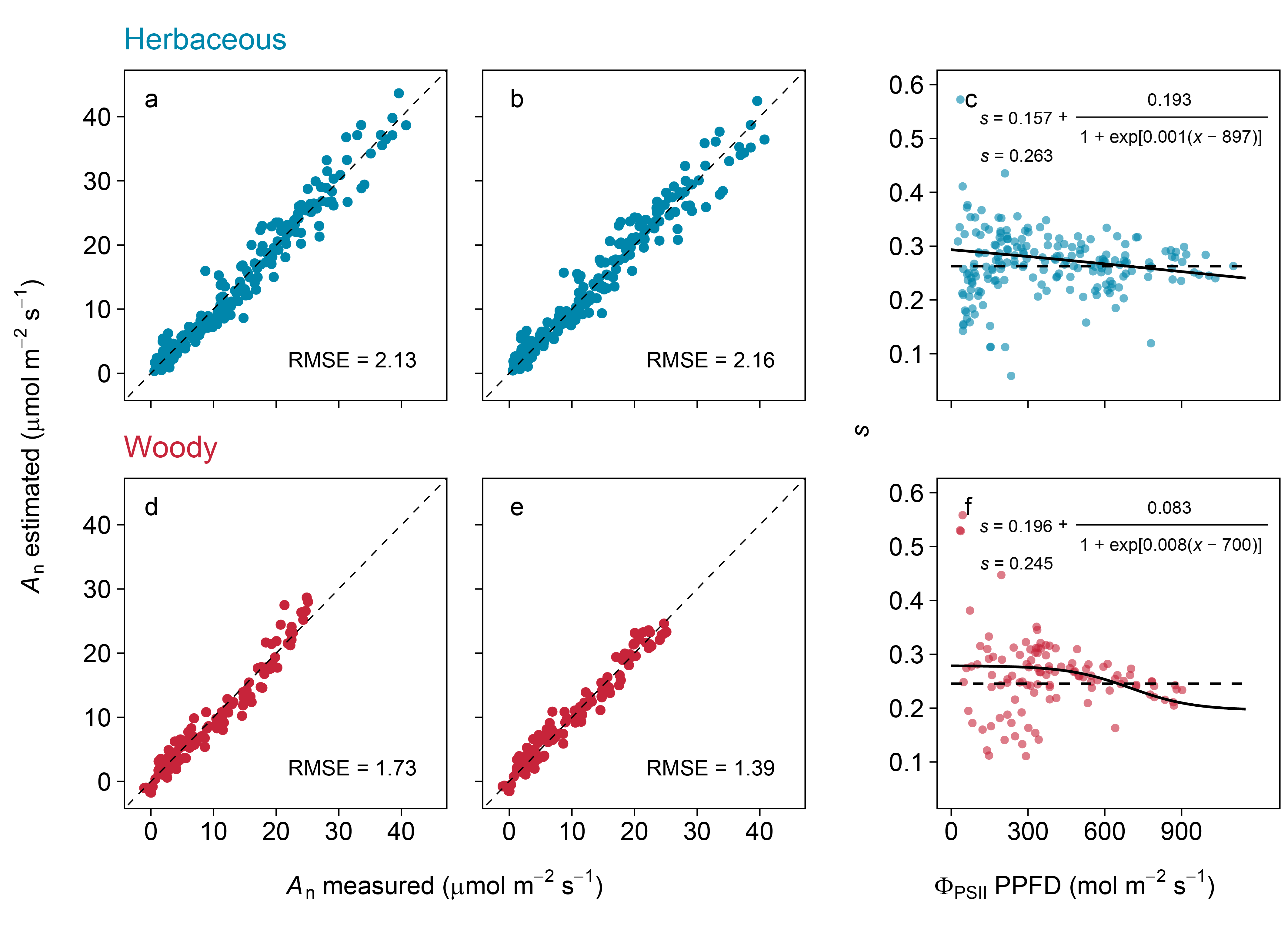


# Figure S4. Relationship between the estimated and measured leaf CO_2_ assimilation rates (*A*_n_) when cross-validation described in the main manuscript was performed using datasets divided into herbaceous and woody species. *A*_n_ was estimated using parameter *s* in Equation (3) calibrated under the assumption that (a, d) *s* is constant and (b, e) *s* varies with quantum yield of photochemistry in PSII (Φ_PSII_) × photosynthetic photon flux density (PPFD). Responses of *s* to Φ_PSII_ PPFD in herbaceous (c) and woody (f) species are also shown. *s* was calculated from Equations (2) and (3) by substituting the measured values of *A*_n_, *C*_i_, Φ_PSII_, and PPFD and the estimated values of Γ^*^ and *R*_d_ from Equations (6)–(8). The regression lines and fitted equations assuming that *s* is constant (dashed line) and that *s* varies with Φ_PSII_PPFD (solid line) are also shown.

Individual calibration provided more reasonable estimates for woody species and equivalent estimates for herbaceous species, compared to combined calibration (i.e., Fig.5 in the main manuscript). Moreover, improvement using variable *s* was more significant for woody species than herbaceous species. These different responses in herbaceous and woody plants resulted in different optimal parameters of *s*. These different parameters were probably derived from the different leaf anatomies between woody and herbaceous plants, which resulted in different intra-leaf light environments and photochemical properties.


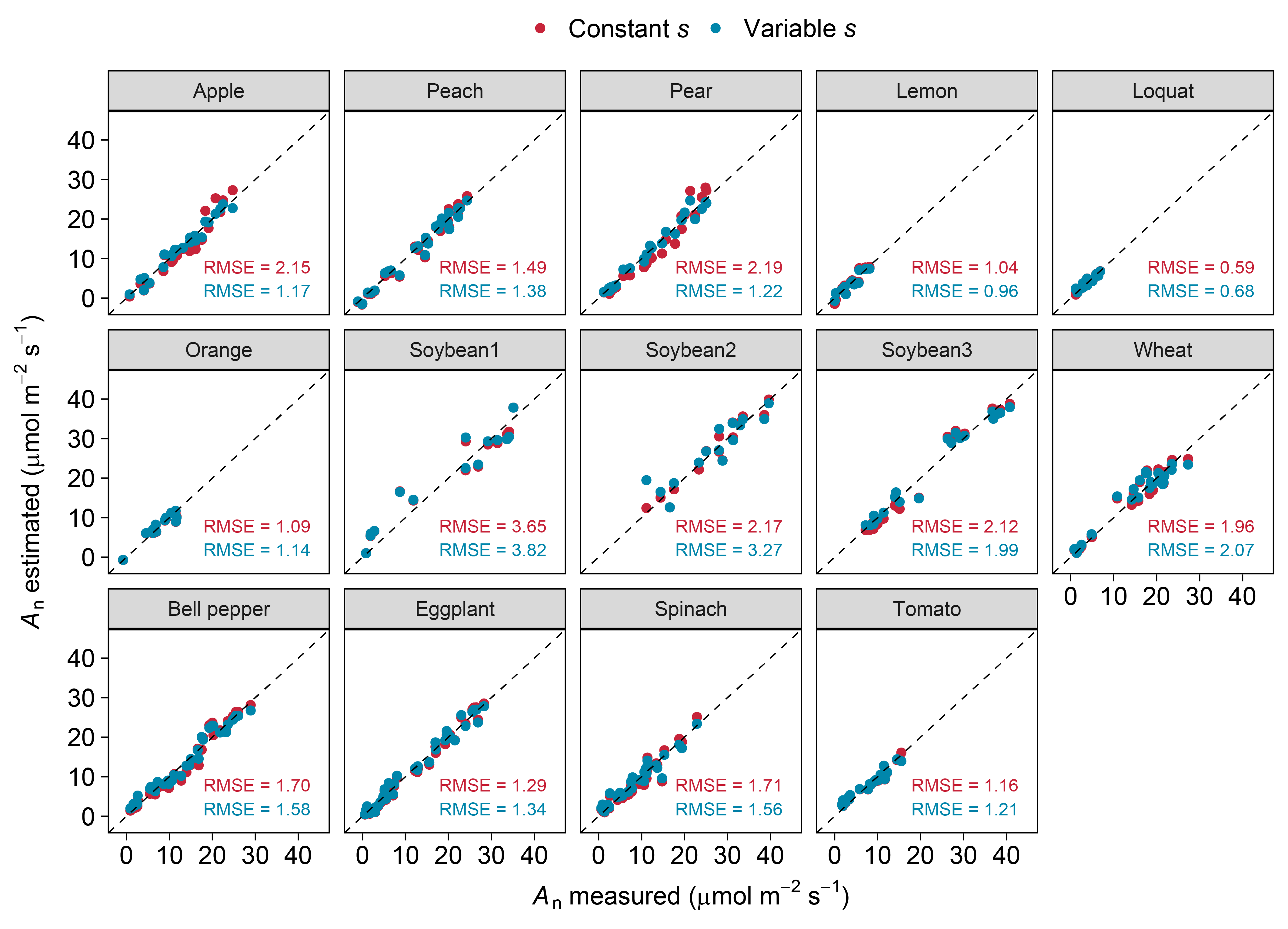


# Figure S5. Relationship between the estimated and measured leaf CO_2_ assimilation rates (*A*_n_) when leave-one-out cross-validation for each species and cultivar was performed. *A*_n_ was estimated using parameter *s* in Equation (3) calibrated under the assumption that (red) *s* is constant and (blue) *s* varies with quantum yield of photochemistry in PSII (Φ_PSII_) × photosynthetic photon flux density (PPFD). The fitted values of *s* were listed in Table S1.

As is the case of the Individual calibration for herbaceous and woody species (Fig. S4), improvement using variable *s* was more significant for woody species than herbaceous species.


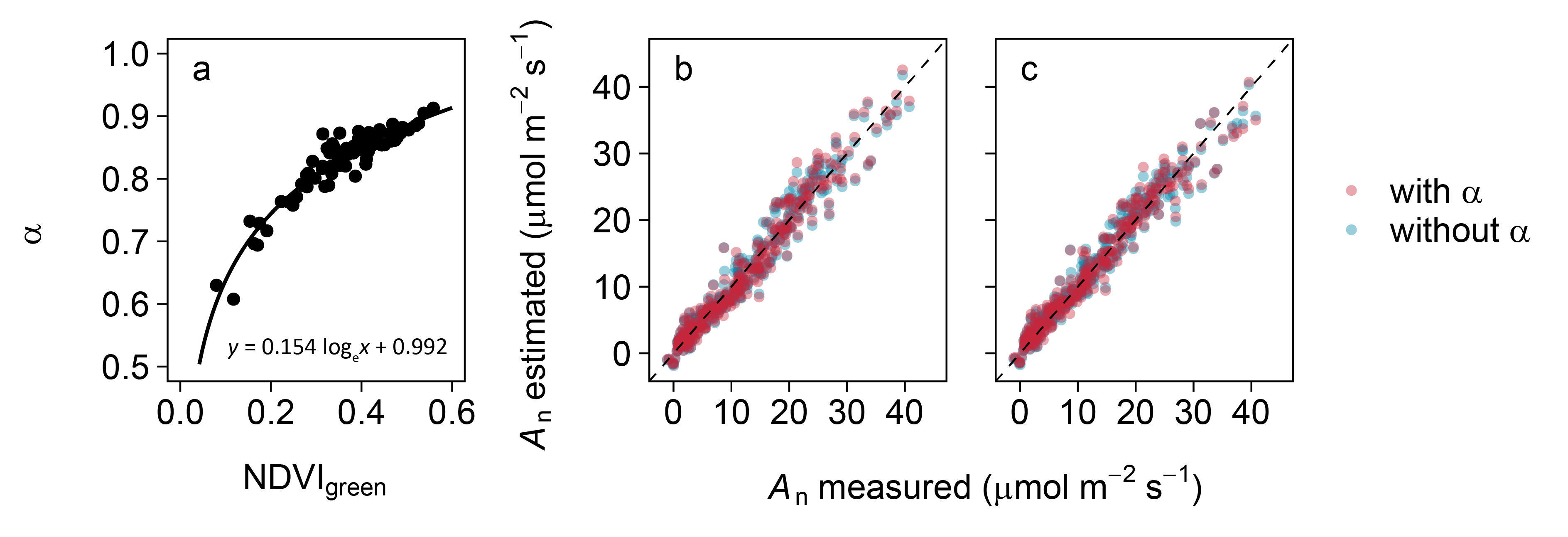


# Figure S6. (a) Relationship between leaf absorptance to photosynthetic photon flux density (*α*) and green normalized difference vegetation index (NDVI_green_). NDVI_green_ was calculated as (*ρ*_750_ – *ρ*_550_) / (*ρ*_750_ + *ρ*_550_), where *ρ*_750_ and *ρ*_550_ are the leaf reflectance at 750 nm and 550 nm, respectively. (b, c) Relationship between estimated and measured leaf CO_2_ assimilation rates (*A*_n_). *A*_n_ was estimated with *α* derived from NDVI_green_ (red) and without *α* estimates (blue). The results of cross-validation with (b) the constant calibrated parameter *s* and (c) variable *s* as a function of quantum yield of photochemistry in PSII (Φ_PSII_) × photosynthetic photon flux density (PPFD) are shown.

*α* and NDVI_green_ (*ρ*_750_ and *ρ*_550_) were measured using a leaf spectrometer (CI‐710s, CID Bio‐Science) in six species (wheat, soybean, spinach, eggplant, tomato, and bell pepper) to obtain their relationship. For *A*_n_ estimates, NDVI_green_ was measured in 12 species using a leaf spectrometer (PolyPen RP 410, Photon Systems Instruments).

In the present study, *α* values estimated from NDVI_green_ ranged from 0.79 to 0.92, with a mean value of 0.86 in 12 species (n = 310), which is the typical value for healthy leaves. Independent estimation of *α* could not greatly improve *A*_n_ estimate, indicating that assuming the constant value of *α* for healthy leaves is a reasonable strategy for high-throughput phenotyping in the field.


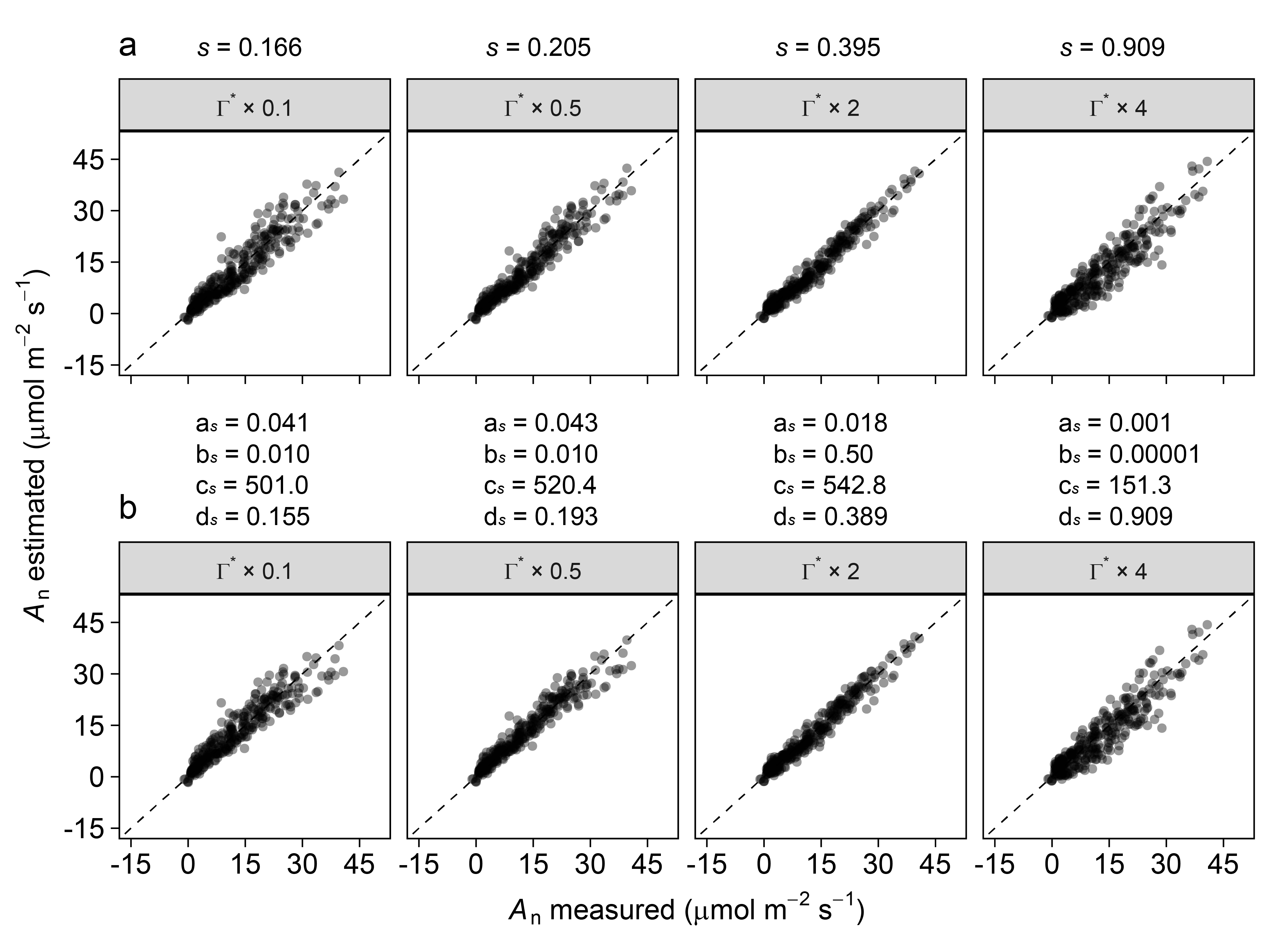


# Figure S7. Relationship between estimated and measured leaf CO_2_ assimilation rates (*A*_n_). *A*_n_ was estimated with CO_2_ compensation point in the absence of day respiration (Γ^*^) changing from its default values to –90% to 400%, with other variables and parameters set to the measured or estimated values described in the main manuscript. The results of cross-validation with (a) the constant calibrated parameter *s* and (b) variable *s* as a function of quantum yield of photochemistry in PSII (Φ_PSII_) × photosynthetic photon flux density (PPFD) are shown. The calibrated parameter values for *s* are also shown above each figure.

Although an extremely overestimated or underestimated Γ^*^ increased an error in *A*_n_ estimate, a dire error in *A*_n_ could be avoided when *s* was calibrated. However, such large variations in Γ^*^ likely occur in the field (e.g., Γ^*^ at 25 °C ranged from 12 to 177 μmol mol^−1^ among 20 crop species; Hermida-Carrera et al., 2016); thus, appropriate Γ^*^ values should be input in the practical use of the porometer-fluorometer method.


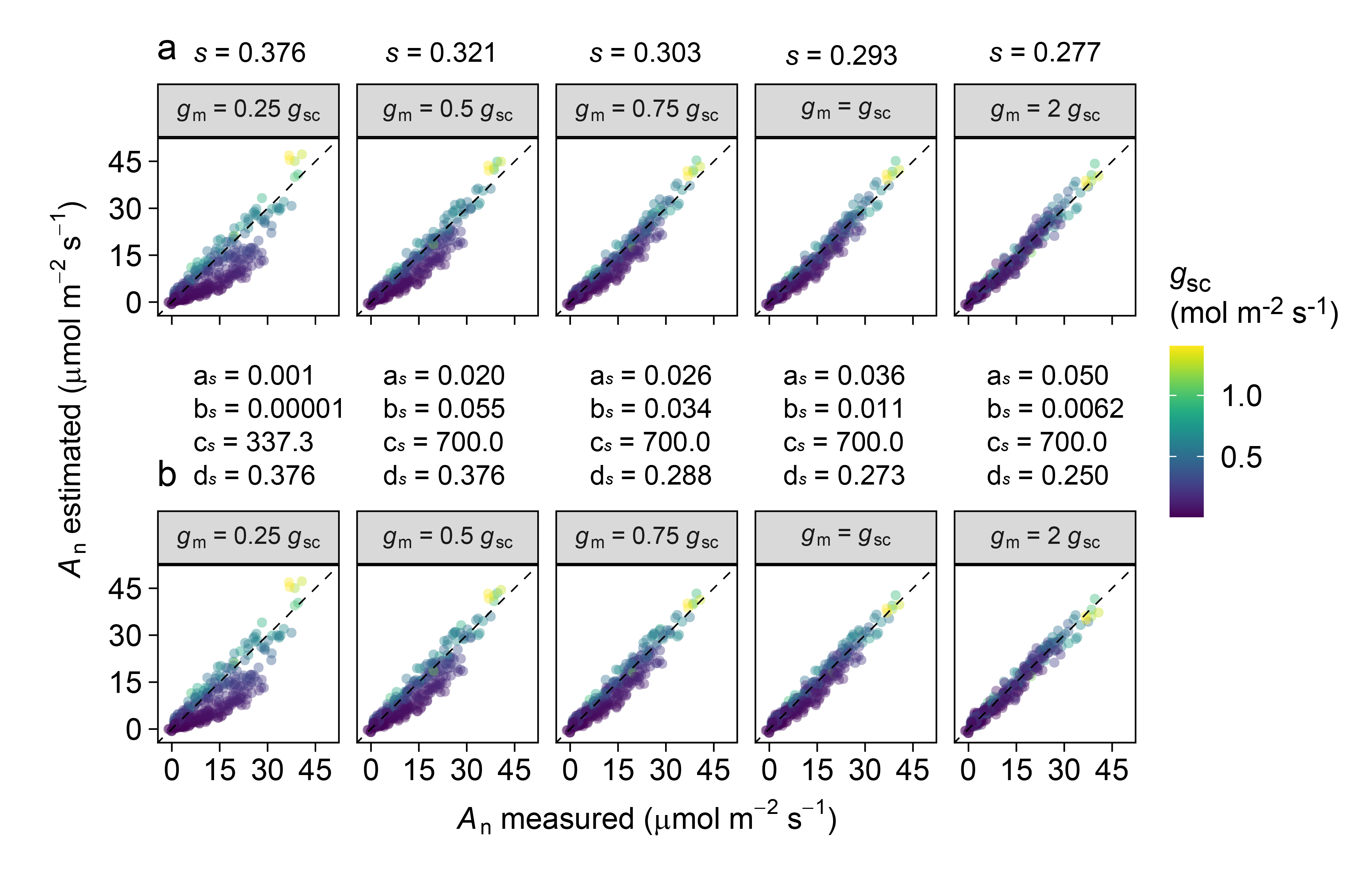


# Figure S8. Relationship between estimated and measured leaf CO_2_ assimilation rates (*A*_n_). *A*_n_ was estimated with mesophyll conductance (*g*_m_) assumed to be proportional to stomatal conductance (*g*_sc_), and other variables and parameters were set to the measured or estimated values described in the main manuscript. The results of cross-validation with (a) the constant calibrated parameter *s* and (b) variable *s* as a function of quantum yield of photochemistry in PSII (Φ_PSII_) × photosynthetic photon flux density (PPFD) are shown. The calibrated parameter values for *s* are also shown above each figure.

Calibration of *s* successfully estimated *A*_n_ when *g*_m_ was comparable to *g*_sc_ or higher than *g*_sc_. This indicates that the porometer-fluorometer method is applicable as long as such conditions are fulfilled, even if actual *g*_m_ is considerably low. In contrast, an error in *A*_n_ estimate increased when *g*_m_ was considerably lower than *g*_sc_ even if *s* was calibrated. In particular, the error increased when absolute values of *g*_sc_ were low. These results indicate that *g*_m_ should be individually determined or calibrated under the conditions that an absolute value of *g*_m_ is low and further that value is lower than *g*_sc_**.**


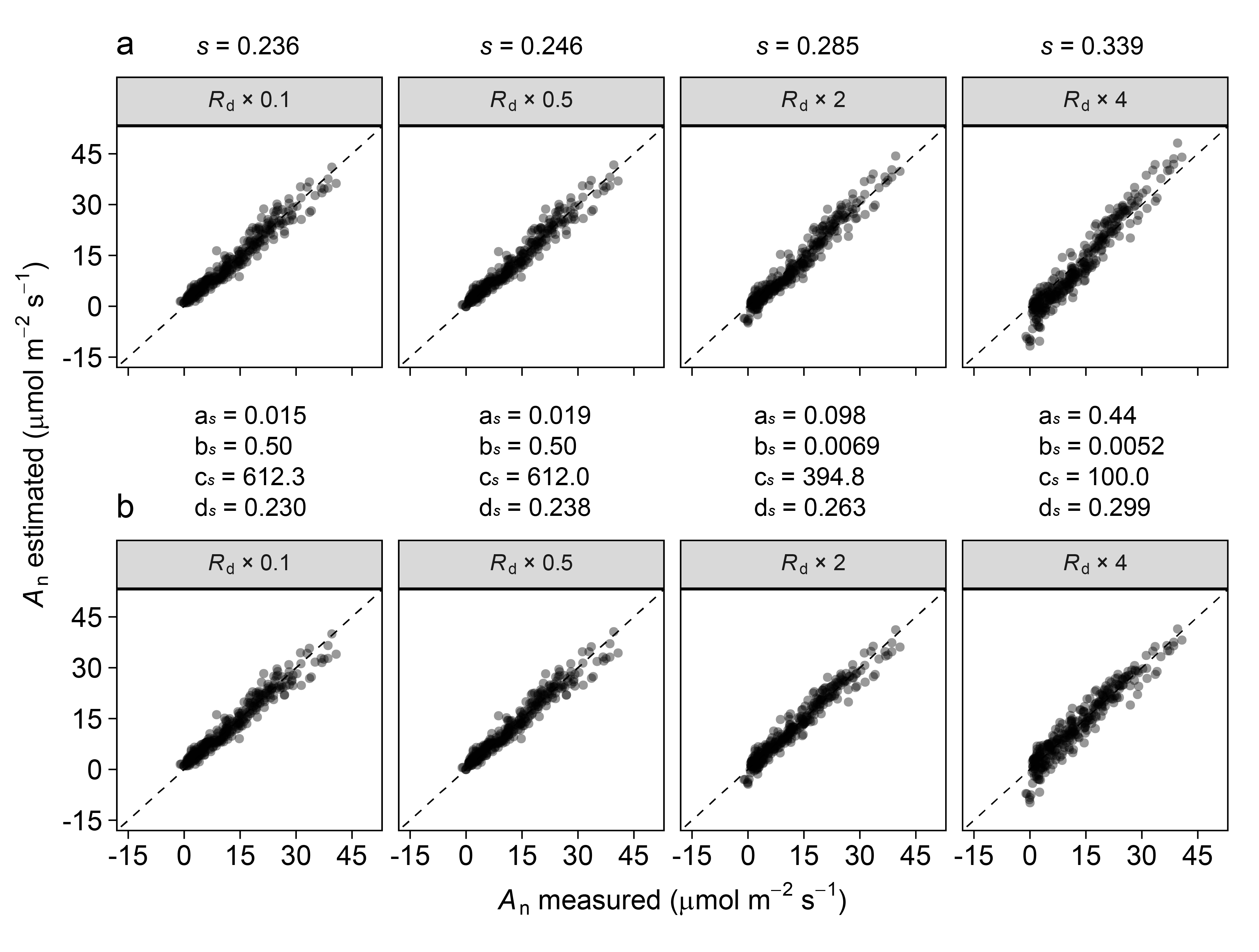


# Figure S9. Relationship between estimated and measured leaf CO_2_ assimilation rates (*A*_n_). *A*_n_ was estimated with day respiration rate (*R*_d_) changing from its default value to –90% to 400%, with other variables and parameters set to the measured or estimated values described in the main manuscript. The results of cross-validation with (a) the constant calibrated parameter *s* and (b) variable *s* as a function of quantum yield of photochemistry in PSII (Φ_PSII_) × photosynthetic photon flux density (PPFD) are shown. The calibrated parameter values for *s* are also shown above each figure.

An extremely overestimated *R*_d_ resulted in significant error in *A*_n_ estimate at a low *A*_n_, where the relative contribution of *R*_d_ is large. This error cannot be corrected by calibrating the parameter *s*, which is the slope in Equation (2) shown in the main manuscript, because *R*_d_ is the intercept.

# Table S1. The fitted values of the parameter *s* for each species and cultivar. *s* in Equation (3) was calibrated under the assumption that *s* is constant and *s* varies with quantum yield of photochemistry in PSII (Φ_PSII_) × photosynthetic photon flux density (PPFD). The calibration of *s* was performed by minimizing the root mean square error (RMSE) between the estimated and observed values of *A*_n_ using differential evolution with the DEoptim package in R.

|  | Constant *s* |  | Variable *s* | | | |
| --- | --- | --- | --- | --- | --- | --- |
| Species and cultivar | *s* |  | a*_s_* | b*_s_* | c*_s_* | d*_s_* |
| Apple | 0.247 |  | 0.102 | 0.011 | 702.8 | 0.188 |
| Peach | 0.248 |  | 0.033 | 0.500 | 543.2 | 0.237 |
| Pear | 0.238 |  | 0.098 | 0.007 | 633.0 | 0.198 |
| Lemon | 0.295 |  | 0.171 | 0.500 | 202.8 | 0.272 |
| Loquat | 0.161 |  | 0.030 | 0.423 | 80.7 | 0.160 |
| Orange | 0.274 |  | 0.057 | 0.500 | 406.1 | 0.219 |
| Soybean1 | 0.284 |  | 0.036 | 0.500 | 753.8 | 0.254 |
| Soybean2 | 0.245 |  | 0.022 | 0.500 | 862.1 | 0.240 |
| Soybean3 | 0.265 |  | 0.041 | 0.270 | 598.5 | 0.260 |
| Wheat | 0.245 |  | 0.038 | 0.500 | 524.8 | 0.232 |
| Bell pepper | 0.262 |  | 0.058 | 0.500 | 411.1 | 0.253 |
| Eggplant | 0.278 |  | 0.019 | 0.500 | 436.8 | 0.272 |
| Spinach | 0.272 |  | 0.049 | 0.072 | 274.7 | 0.255 |
| Tomato | 0.290 |  | 0.042 | 0.500 | 352.5 | 0.252 |

Descriptions of a*_s_*, b*_s_*, c*_s_*, and d*_s_* are described in the main manuscript.

Although the fitted *s* showed species- and cultivar-dependent values, the coefficient of variation (CV) of *s* was 12% and significantly lower than those of the parameters of the conventional empirical models shown in Fig. S3. We note that large variations in b*_s_* and c_s_ are partly derived from a little data point for each species and cultivar, and additional sampling is required to obtain reliable values. Moreover, *s* values likely vary with environmental conditions, even in the same species and cultivar. Direct use of the above values without any calibration should be avoided.

# Note S1 Analytical solution of the model for estimating *A*_n_.

*A*_n_ can be analytically obtained by combining the biochemical photosynthesis model with the equations for electron transport and CO_2_ transport (Kitao et al., 2021):

|  | $\text{​}\text{A}_{\text{n}}\text{ = }\frac{-\sqrt{\text{a}\text{ }\text{g}_{\text{tc}}^{\text{2}}+\text{b} \text{g}_{\text{tc}}+\text{c}}\text{ + }\text{d} \text{g}_{\text{tc}}+\text{e}}{\text{8}}$ |  |
| --- | --- | --- |
|  | $\text{​}\text{a}\text{ = 64} \text{Γ}^{\text{*}\text{2}}+{\text{64}\text{ }\text{C}}_{\text{a}}\text{Γ}^{\text{*}}+\text{16}\text{ }\text{C}_{\text{a}}^{\text{2}}$ |  |
|  | $\text{​}\text{b}\text{ = }\left( \text{64} \text{R}_{\text{d}}+\text{32}\text{ }\text{J} \right)\text{Γ}^{\text{*}}+{\text{32 }\text{C}}_{\text{a}}\text{R}_{\text{d}}-\text{8 }\text{C}_{\text{a}}\text{ }\text{J}$ |  |
|  | $\text{​}\text{c}\text{ }\text{= 16}{\text{ }\text{R}}_{\text{d}}^{\text{2}}-\text{8 }\text{R}_{\text{d}}\text{ }\text{J}+\text{J}^{\text{ }\text{2}}$ |  |
|  | $\text{​}\text{d}\text{ }\text{= 8 }\text{Γ}^{\text{*}}+{\text{4}\text{ }\text{C}}_{\text{a}}$ |  |
|  | $\text{​}\text{e}\text{ }\text{= }-\text{4}\text{ }\text{R}_{\text{d}}+\text{J}$ |  |

The variables are described in the main manuscript.

# Note S2 Growth conditions of plant materials.

Wheat (*Triticum aestivum* cv. Satonosora), soybeans (*Glycine max*, cv. Enrei, Soramizuki, and UA4910, described as Soybean1, Soybean2, and Soybean3 in the main manuscript) were grown in experimental fields at the Institute for Agro-Environmental Sciences, National Agriculture and Food Research Organization, Tsukuba, Ibaraki, Japan. Wheat was cultivated from November 2023 to May 2024. Soybean was cultivated from 11 June 2024 to 28 October 2024 for ‘Soramizuki’ , from 24 July 2024 to 10 October 2024 for ‘Enrei’, and from 24 July 2024 to 3 November 2024 ‘UA4910.’ Both crops were grown using the conventional fertilizer management and planting densities recommended for the study site. The mean temperature, mean relative humidity, mean total daily solar radiation, and total precipitation at this site during the wheat growing period were 9.3°C, 68.8 %, 12.6 MJ m^−2^, and 400 mm, respectively. The corresponding values during the soybean-growing period were 25.2°C, 80.1 %, 16.4 MJ m^−2^, and 574 mm for ‘Soramizuki’, 26.4°C, 80.5 %, 16.5 MJ m^−2^, and 325 mm for ‘Enrei’, and 24.5°C, 80.4 %, 15.0 MJ m^−2^, and 429 mm for ‘UA4910’.

Tomato (*Solanum lycopersicum*, cv. Momotaro peace), eggplant (*Solanum melongena*, cv. Ryoma), bell pepper (*Capsicum annuum*, cv. Makarin), spinach (*Spinacia oleracea* cv. Aggressive) were grown in experimental greenhouses of the IoP (Internet of Plants) Collaborative Creation Center at Kochi University, Nankoku, Kochi, Japan. Tomatoes were cultivated from August 17, 2023, to June 27, 2024, eggplant from September 1, 2023, to June 19, 2024, and bell peppers from September 21, 2023, to July 26, 2024. Tomato and bell peppers were cultivated with hydroponic culture systems, and a conventional nutrient solution recommended for the study site was supplied. Eggplant was cultivated with soil culture systems, and water and nutrient supply were performed using sprinkler hoses placed at the center of the ridges. Spinach was cultivated from December 2023 to May 2024 in nursery pots in the greenhouse. Top and side ventilation windows and heaters were operated to maintain the temperature in the greenhouses. CO_2_ generators were operated to maintain daytime CO_2_ concentrations above 400 μmol mol^−1^. The mean temperature, mean relative humidity, and mean total daily solar radiation during the tomato-growing period in the greenhouses were 18.7°C, 82.2%, and 7.9 MJ m^−2^, respectively. The corresponding value during the eggplant-growing period was 19.9°C, 86.2 %, 8.7 MJ m^−2^. The corresponding values during the bell pepper growth period were 17.7°C, 82.2 %, 7.7 MJ m^−2^. The corresponding value during the spinach-growing period was 15.6°C, 84.7 %, 6.8 MJ m^−2^.

Apple (*Malus domestica*, cv. Fuji), peach (*Prunus persica* cv. Sakuhime), and pear (*Pyrus pyrifolia* cv. Kosui), loquat (*Rhaphiolepis bibas*, cv. Mogi), lemon (*Citrus limon*, cv. Rinoka), and orange (*Citrus unshiu*, cv. Okitsuwase) were grown in the experimental fields at the Institute of Fruit Tree and Tea Science, National Agriculture and Food Research Organization, Tsukuba, Ibaraki, Japan. These trees were planted in 20-L pots filled with nursery soil in 2008 for apples, 2018 for peaches, 2007 and 2009 for pears, 2015 for loquats, 2018 for lemons, and 2002 for oranges. Water was supplied using sprinkler hoses placed in pots. The annual mean temperature, relative humidity, and solar radiation that the trees experienced were 15.2°C, 76 %, and 14.0 MJ m^−2^, respectively, and the annual total precipitation was 1374 mm, based on average values from 2019 to 2023. We note that these trees likely restricted their growth because of the capacity of 20-L pots and the fact that they were grown in a suboptimal environment.

# Reference

Ball JT, Woodrow IE, Berry JA. 1987. A model predicting stomatal conductance and its contribution to the control of photosynthesis under different environmental conditions. In: Biggins I, eds. *Progress in photosynthesis research*. Dordrecht, NLD: Springer Dordrecht, 221–224. <https://doi.org/10.1007/978-94-017-0519-6_48>

Hermida-Carrera C., Kapralov M.V., Galmés J. 2016. Rubisco catalytic properties and temperature response in crops. *Plant Physiology* **171**, 2549–2561. <https://doi.org/10.1104/pp.16.01846>

Kitao, M., Yasuda, Y., Kodani, E., Harayama, H., Awaya, Y., Komatsu, M. et al. 2021. Integration of electron flow partitioning improves estimation of photosynthetic rate under various environmental conditions based on chlorophyll fluorescence. *Remote Sensing of Environment* **254**, 112273. <https://doi.org/10.1016/j.rse.2020.112273>
